# Disclosing common biological signatures and predicting new therapeutic targets in schizophrenia and obsessive–compulsive disorder by integrated bioinformatics analysis

**DOI:** 10.1186/s12888-023-04543-z

**Published:** 2023-01-14

**Authors:** Abdolhakim Ghanbarzehi, Ali Sepehrinezhad, Nazanin Hashemi, Minoo Karimi, Ali Shahbazi

**Affiliations:** 1 Department of Neuroscience, Faculty of Advanced Technologies in Medicine, Iran University of Medical Sciences, Tehran, Iran; 2 Neuroscience Research Center, Mashhad University of Medical Sciences, Mashhad, Iran; 3 Cellular and Molecular Research Center, Iran University of Medical Sciences, Tehran, Iran; 4 Department of Biotechnology, Bangalore University, Bangalore, Karnataka India; 5 Department of Audiology, School of Rehabilitation, Tehran University of Medical Sciences, Tehran, Iran

**Keywords:** Schizophrenia, Obsessive–compulsive disorder, MicroRNA, Common mechanisms, Drug repurposing, Bioinformatics approach

## Abstract

Schizophrenia (SCZ) is a severe mental illness mainly characterized by a number of psychiatric symptoms. Obsessive–compulsive disorder (OCD) is a long-lasting and devastating mental disorder. SCZ has high co-occurrence with OCD resulting in the emergence of a concept entitled “schizo-obsessive disorder” as a new specific clinical entity with more severe psychiatric symptoms. Many studies have been done on SCZ and OCD, but the common pathogenesis between them is not clear yet. Therefore, this study aimed to identify shared genetic basis, potential biomarkers and therapeutic targets between these two disorders. Gene sets were extracted from the Geneweaver and Harmonizome databases for each disorder. Interestingly, the combination of both sets revealed 89 common genes between SCZ and OCD, the most important of which were *BDNF, SLC6A4, GAD1, HTR2A, GRIN2B, DRD2, SLC6A3, COMT, TH* and *DLG4*. Then, we conducted a comprehensive bioinformatics analysis of the common genes. Receptor activity as the molecular functions, neuron projection and synapse as the cellular components as well as serotonergic synapse, dopaminergic synapse and alcoholism as the pathways were the most significant commonalities in enrichment analyses. In addition, transcription factor (TFs) analysis predicted significant TFs such as HMGA1, MAPK14, HINFP and TEAD2. Hsa-miR-3121-3p and hsa-miR-495-3p were the most important microRNAs (miRNAs) associated with both disorders. Finally, our study predicted 19 existing drugs (importantly, Haloperidol, Fluoxetine and Melatonin) that may have a potential influence on this co-occurrence. To summarize, this study may help us to better understand and handle the co-occurrence of SCZ and OCD by identifying potential biomarkers and therapeutic targets.

**Supplementary Information:**

The online version contains supplementary material available at 10.1186/s12888-023-04543-z.

## Introduction

Schizophrenia (SCZ) is a complex and severe mental illness mainly characterized by a combination of positive symptoms (delusions and hallucinations), negative symptoms (social withdrawal, paucity of spontaneous speech and amotivation) and debilitating cognitive deficits [1]. Overall, the prevalence of SCZ in both sexes is approximately equal, but with earlier onset and greater severity in males than in females [2]. Environmental and social factors such as childhood trauma and social isolation predispose individuals to SCZ [3, 4]. However, it is highly heritable (~ 80%) with complex polygenic architecture which both common and rare genetic variants contribute to its etiology [5]. Emerging evidence has suggested that SCZ has a common molecular etiology with other psychiatric disorders such as obsessive–compulsive disorder (OCD), autism spectrum disorders (ASD) and bipolar disorder (BD), despite their unique clinical characteristics [6].

OCD, with the prevalence of about 1–3% in the general population, is a long-lasting and devastating mental disorder characterized by recurrent, persistent and unwanted thoughts, images or impulses called obsessions and repetitive behaviors called compulsions [7]. The World Health Organization has ranked it as one of the most debilitating disorders worldwide as it results in poor quality of life and can substantially impair the patient’s occupational, marital, emotional and social functioning [8, 9]. Moreover, plenty of epidemiological studies demonstrated that co-occurrence rates in OCD are generally higher than rates of other comorbid disorders [10–12].

In the past years, the categorizing of psychiatric disorders has been rearranged through the increasing a lot of studies that emphasize co-occurring and/or comorbid disorders [13]. Relating to SCZ, the specific co-occurrence of obsessive–compulsive symptoms (OCS) and SCZ has been revealed for more than a century (in 1878) [14]. A meta-analysis study indicated that nearly 38.3% of SCZ patients experience anxiety disorders during their illnesses. Also, the prevalence of OCD in these patients was reported at 12.1% [15]. In the same way, another meta-analysis reported that the prevalence of OCS and OCD in SCZ patients is 30.3% and 12.3%, respectively [16]. These results along with abundant research that focus on the presence of OCD and OCS among the SCZ patients emerged the concept of “schizo-obsessive disorder” as a new specific clinical entity [17–19]. Meanwhile, the accurate examination of these patients manifested that they have more severe psychotic and depressive symptoms, lower social functioning and higher suicidality [13]. Although the major psychiatric disorders are very debilitating, early diagnosis and treatment can substantially reduce the unfavorable outcomes of them [7]. Therefore, identifying more possible biomarkers and effective drugs can be essential for reducing the severity of these disorders.

There are two hypotheses for the co-occurrence of OCS during the course of SCZ. One assumes that second-generation antipsychotics, especially clozapine, might exacerbate or generate second-onset OCS. The second is an important role of genetic risk factors that dispose of patients with SCZ to develop OCS [20]. In the past decade, studies using both gene data and genome-wide association study (GWAS) have debated that some genes may be responsible for the co-occurrence of SCZ and OCS/OCD. [20–22]. However, as far as we know, there has been no bioinformatic study performed with a special focus on the common genes between these disorders. In a recent study, O’Connell et al. displayed common genetic etiology for SCZ, BD, ASD and OCD. They proposed that more research on shared components of these disorders is needed to obtain actionable and translatable results in order to combat psychiatric disorders [6].

Although there have been many studies done on SCZ and OCD, the common pathogenesis of them is not been well identified at the molecular level until now. Based on this point, we assume that the co-occurrence of SCZ and OCD is rooted in the genetic similarities and it may reveal the shared genetic basis, potential biomarkers and therapeutic targets between the two disorders. In this regard, we conducted a comprehensive bioinformatics analysis to identify common genes, molecular functions, cellular components and biological pathways along with predicting transcription factors (TFs) and posttranscriptional regulator microRNAs (miRNAs) as well as to repurpose candidate drugs for both SCZ and OCD.

## Material and Methods

### Finding related genes and construction of genetic network

At the beginning of conducting the study, the existing data were used in this way that all genes related to SCZ and OCD were extracted from GeneWeaver (https://www.geneweaver.org/) and Harmonizome (https://maayanlab.cloud/Harmonizome/) databases [23, 24]. GeneWeaver is an available web-based tool for conducting integrative functional genomics on our target genes in combination with large gene sets from different databases [25]. Harmonizome is a collection of processed datasets that provides comprehensive information about genes and proteins concerning diseases [26] and contained 71,597,788 associations between 295,485 attributes and 56,720 genes from 112 datasets provided by 65 resources. Extracted gene sets were taken from genome-wide association studies (GWAS) and other genetic association studies in Harmonizome. After that, each separated gene set was pasted into an excel file and then their common genes were saved for bioinformatics analyses. All shared genes were uploaded into the STRING (https://string-db.org/) to obtain gene–gene interactions file [27]. Finally, we used windows version of Cytoscape 3.7.0 to visualize and interpret gene–gene interactions and reconstruct the genetic network for shared genes between SCZ and OCD [28]. Besides, Network Analyzer Tool was applied to calculate and identify network parameters such as degree and betweenness centrality. In all conducted analyses* P*-value < 0.05 or false discovery rate (FDR) < 0.05 were considered as significantly level.

### Gene Ontology and pathway enrichment analysis

In order to investigate the possible mechanisms of OCD in SCZ, we conducted a gene enrichment analysis through ToppGene (https://toppgene.cchmc.org/). To achieve this goal, we uploaded common genes into the ToppGene through ToppFun section and then selected Gene Ontology (GO) to proceed the enrichment analysis for Molecular Function and Cellular Component of target genes. Also, our target genes were submitted into the KEEG pathway section of ToppGene database to investigate the possible disrupted pathways in both SCZ and OCD [29, 30]. Statistical significance (*p* < 0.05) was considered by a likelihood-ratio test with correction for FDR using Benjamini–Hochberg method to show multiple comparison.

### Prediction of transcription factors and microRNAs besides drug repurposing

We used Enrichr (https://maayanlab.cloud/Enrichr/) to predict some significant TFs for common genes between SCZ and OCD. This goal was achieved through TRANSFAC and JASPAR PWMs panel of Enrichr [31]. In addition, miRDB database (http://mirdb.org/index.html) was used to predict main target miRNAs that represented both SCZ and OCD [32]. miRDB database uses constructive machine learning procedures to find target prediction score between 50–100 throughout datasets. Higher target prediction score represented more validated miRNA. All human miRNAs with target score of more than 95, were selected and uploaded to Cytoscape software to visualize gene-miRNAs network.

Finally, drug repurposing was conducted through Stitch and CTD section of ToppGene database. Cytoscape was used to visualize gene-pathways, gene-miRNAs, gene-drug interaction networks and determine the degree parameter in each network.

## Results

### Identification of SCZ-OCD shared genes and finding hub genes from reconstructed genetic network

**Fig. 1:**
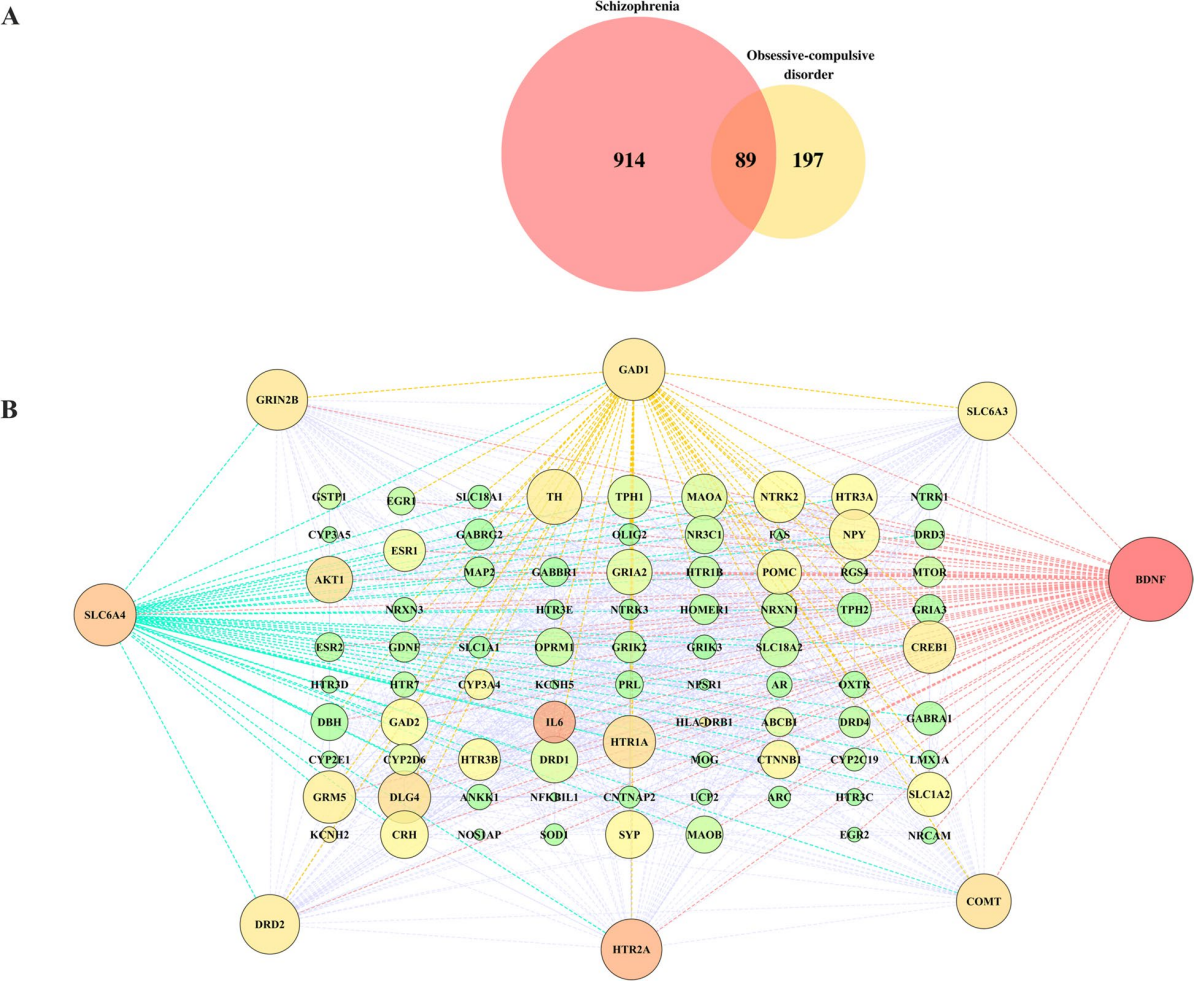
The genetic network of common genes between SCZ and OCD. (A) a Venn diagram displaying the number of genes over both SCZ and OCD. (B) Genetic network of SCZ-OCD associated genes consists of 89 nodes that size and color adjusted according to degree and betweenness centrality respectively to specify hub genes. Larger nodes have a higher degree and Red nodes have a higher betweenness centrality. Eight nodes with higher degree are located in the outer sides. The main features of the network are included: clustering coefficient:0.584; network diameter:5; network radius:3; network centralization: 0.478; network density:0.249

At first, with the exploration of existing data in Geneweaver and Harmonizome, we identified 914 and 197 genes in association with SCZ and OCD respectively (Supplementary Tables 1 and 2). Interestingly, the combination of both gene sets revealed that 89 genes were shared between these two disorders (Fig. 1 and Supplementary Table 3). The following analyzing of common genetic network manifested that ten genes such as brain-derived neurotrophic factor (*BDNF*; Degree:63; Betweenness centrality:0.104), sodium-dependent serotonin transporter and solute carrier family 6 member 4 (*SLC6A4*; D:46; B:0.050), glutamate decarboxylase 1 (*GAD1*; D:46; B:0.026), 5-hydroxytryptamine receptor 2A (*HTR2A*; D:45; B:0.059), glutamate ionotropic receptor NMDA type subunit 2B (*GRIN2B*; D:45; B:0.027), dopamine receptor D2 (*DRD2*; D:44; B:0.024), solute carrier family 6 member 3 (*SLC6A3*; D:43; B:0.020), catechol-O-methyltransferase (*COMT*; D:40; B;0.032), tyrosine hydroxylase (*TH*; D:40; B:0.022) and discs large MAGUK scaffold protein 4 (*DLG4*; D:38; B:0.033) were the most significant genes (hub genes) according to their network parameters (Fig. 1). To note, all subsequent bioinformatic analyses were conducted on the common genes between SCZ and OCD.

### Gene Ontology and pathway analysis for common genes

**Fig. 2:**
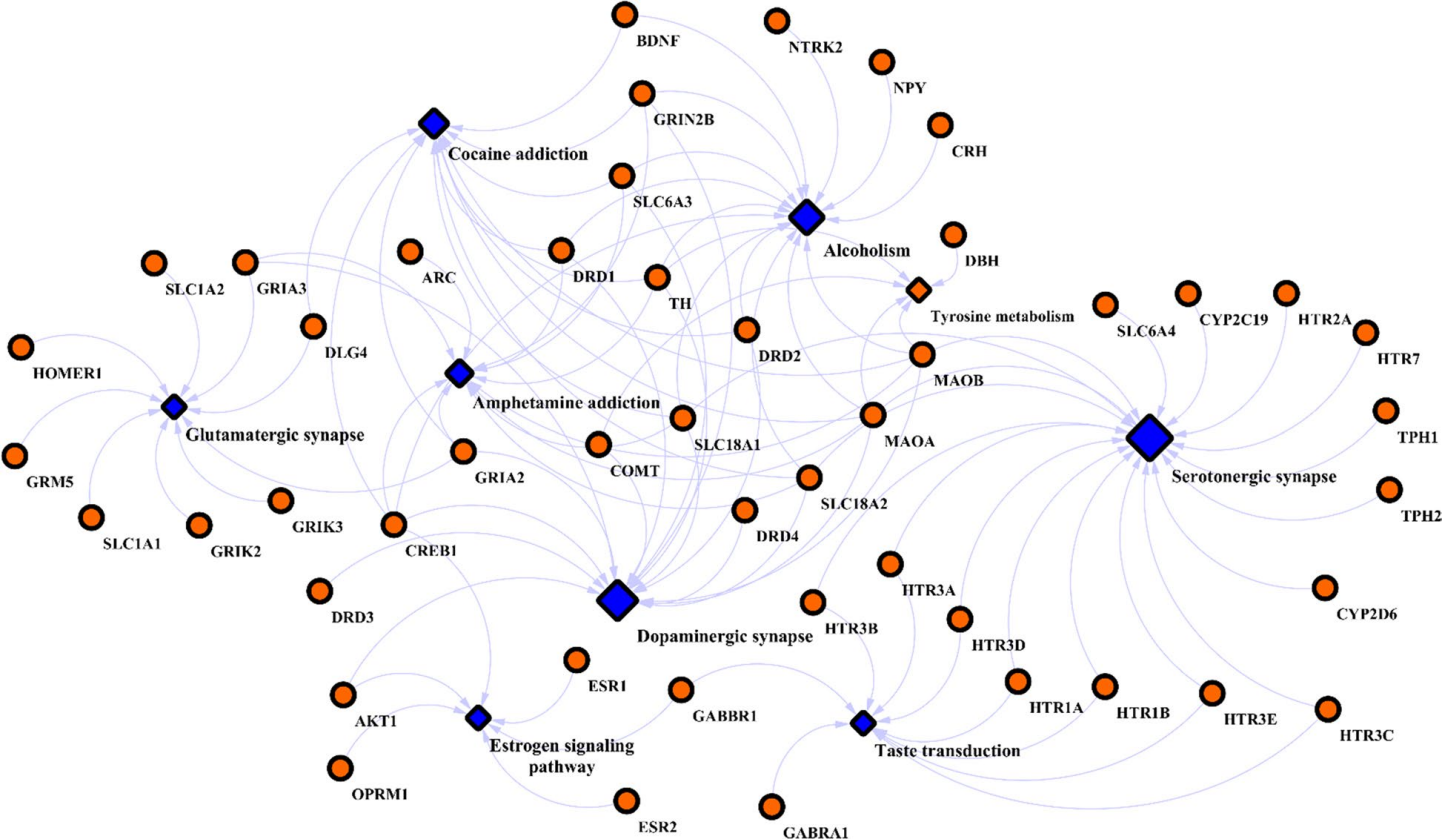
Gene-pathway interaction network (KEGG) for SCZ-OCD shared genes. Each pathway (blue nodes) is connected to its related genes (red nodes) through edges. Pathways that are more connected are represented bigger than others

GO enrichment analyses revealed some significant molecular functions such as neurotransmitter receptor activity, transmitter-gated ion channel activity, signaling receptor activity, molecular transducer activity, postsynaptic neurotransmitter receptor, transmembrane signaling receptor activity, glutamate binding, ligand-gated ion channel activity and dopamine binding that may involve in both SCZ and OCD (Table.1). These analyses also resulted that neuron projection, synapse, somatodendritic compartment, neuronal cell body, dendritic tree, intrinsic component of synaptic membrane, synapse, neuron spine and distal axon were the most disrupted cellular components in these two disorders (Table.1). In addition, KEGG pathway analysis indicated that some pathways such as serotonergic synapse, cocaine addiction, dopaminergic synapse, amphetamine addiction, alcoholism, taste transduction, glutamatergic synapse, tyrosine metabolism and estrogen signaling pathway may contribute to the co-occurrence of OCD in SCZ patients. Reconstructed pathway-gene interaction network indicated that serotonergic synapse (D:18), dopaminergic synapse (D:16), alcoholism (D:14) and cocaine addiction were more connected nodes (Fig. 2).

**Table 1:** Result of molecular function and cellular component enrichment analysis for SCZ-OCD common genes

Name	q-value FDR B&H	Involved Genes
**GO: Molecular Functions**		
Neurotransmitter receptor activity	8.42E-27	GABRG2,DRD1,DRD2,DRD3,HTR1A,DRD4,HTR1B,HTR2A,HTR3A,HTR7,HTR3E, GRIA2,GRIA3,HTR3C,HTR3D,GRIK2,GRIK3,GRIN2B,HTR3B,GRM5,GABBR1,GABRA1
Transmitter-gated ion channel activity	6.02E-15	GABRG2,HTR3A,HTR3E,GRIA2,GRIA3,HTR3C,HTR3D,GRIK2,GRIK3,GRIN2B,HTR3B,GABRA1
Signaling receptor activity	6.02E-15	GABRG2,DRD1,DRD2,DRD3,HTR1A,DRD4,HTR1B,NRXN3,HTR2A,OXTR, HTR3A,NRXN1,HTR7,NTRK1,NTRK2,ESR1,HLA-DRB1,NTRK3,ESR2,NPSR1, HTR3E,GRIA2,GRIA3,HTR3C,HTR3D,GRIK2,GRIK3,GRIN2B,HTR3B,NR3C1,GRM5, FAS,NPY,GABBR1,GABRA1,OPRM1
Molecular transducer activity	6.02E-15	GABRG2,DRD1,DRD2,DRD3,HTR1A,DRD4,HTR1B,NRXN3,HTR2A,OXTR, HTR3A,NRXN1,HTR7,NTRK1,NTRK2,ESR1,HLA-DRB1,NTRK3,ESR2,NPSR1,HTR3E, GRIA2,GRIA3,HTR3C,HTR3D,GRIK2,GRIK3,GRIN2B,HTR3B,NR3C1, GRM5,FAS,NPY,GABBR1,GABRA1,OPRM1
Postsynaptic neurotransmitter receptor activity	6.02E-15	GABRG2,DRD1,DRD2,DRD3,DRD4,GRIA2,GRIA3,GRIK2,GRIK3,GRIN2B, GRM5,GABBR1,GABRA1
Transmembrane signaling receptor activity	8.26E-14	GABRG2,DRD1,DRD2,DRD3,HTR1A,DRD4,HTR1B,HTR2A,OXTR,HTR3A, NRXN1,HTR7,NTRK1,NTRK2,HLA-DRB1,NTRK3,NPSR1,HTR3E,GRIA2, GRIA3,HTR3C,HTR3D,GRIK2,GRIK3,GRIN2B,HTR3B,GRM5, FAS,NPY,GABBR1,GABRA1,OPRM1
Glutamate binding	2.09E-12	GAD1,GAD2,GRIA2,GRIA3,GRIK2,GRIK3,GRIN2B,GRM5,SLC1A1
Ligand-gated ion channel activity	6.77E-12	GABRG2,HTR3A,KCNH2,HTR3E,GRIA2,GRIA3,HTR3C,HTR3D,GRIK2,GRIK3, GRIN2B,HTR3B,GABRA1
Dopamine binding	8.19E-12	SLC6A3,TH,DRD1,DRD2,DRD3,DRD4
Transmembrane transporter activity	8.19E-12	SLC6A3,SLC6A4,GABRG2,DRD4,HTR1B,NRXN3,HTR3A,NRXN1,SLC18A1,SLC18A2,KCNH2,UCP2, HTR3E,GRIA2,GRIA3,HTR3C,HTR3D,GRIK2,GRIK3,GRIN2B,HTR3B,SLC1A1,SLC1A2,NPY,GABRA1, ABCB1,OPRM1,KCNH5
Passive transmembrane transporter activity	8.19E-12	GABRG2,DRD4,HTR1B,NRXN3,HTR3A,NRXN1,KCNH2,HTR3E,GRIA2,GRIA3,HTR3C,HTR3D, GRIK2,GRIK3,GRIN2B,HTR3B,SLC1A1,NPY,GABRA1,OPRM1,KCNH5
Serotonin-gated cation-selective channel activity	5.90E-11	HTR3A,HTR3E,HTR3C,HTR3D,HTR3B
Inorganic molecular entity transmembrane transporter activity	6.33E-11	SLC6A3,SLC6A4,GABRG2,DRD4,HTR1B,HTR3A,SLC18A1,SLC18A2,KCNH2,HTR3E,GRIA2,GRIA3,HTR3C, HTR3D,GRIK2,GRIK3,GRIN2B,HTR3B,SLC1A1,SLC1A2,GABRA1,ABCB1,OPRM1,KCNH5
Ligand-gated cation channel activity	1.04E-10	HTR3A,KCNH2,HTR3E,GRIA2,GRIA3,HTR3C,HTR3D,GRIK2,GRIK3,GRIN2B,HTR3B
Monooxygenase activity	1.09E-10	TH,CYP2C19,CYP2D6,CYP2E1,CYP3A4,CYP3A5,ESR1,TPH2,AKT1,DBH,TPH1
serotonin binding	1.18E-10	SLC6A4,HTR1A,DRD4,HTR1B,HTR2A,HTR3A,MAOA,HTR7
Amine binding	1.71E-10	SLC6A4,HTR1A,DRD4,HTR1B,HTR2A,HTR3A,MAOA,HTR7
Cation transmembrane transporter activity	1.74E-10	SLC6A3,SLC6A4,DRD4,HTR1B,HTR3A,SLC18A1,SLC18A2,KCNH2,HTR3E,GRIA2, GRIA3,HTR3C,HTR3D,GRIK2,GRIK3,GRIN2B,HTR3B,SLC1A1,SLC1A2,OPRM1,KCNH5
Amino acid binding	2.37E-10	GAD1,GAD2,TH,GRIA2,GRIA3,GRIK2,GRIK3,GRIN2B,GRM5,SLC1A1
Ion channel activity	3.41E-10	GABRG2,DRD4,HTR1B,HTR3A,KCNH2,HTR3E,GRIA2,GRIA3,HTR3C,HTR3D,GRIK2,GRIK3, GRIN2B,HTR3B,SLC1A1,GABRA1,OPRM1,KCNH5
Catecholamine binding	4.35E-10	SLC6A3,TH,DRD1,DRD2,DRD3,DRD4
Ion transmembrane Transporter activity	4.95E-10	SLC6A3,SLC6A4,GABRG2,DRD4,HTR1B,HTR3A,SLC18A1,SLC18A2,KCNH2,HTR3E,GRIA2, GRIA3,HTR3C,HTR3D,GRIK2,GRIK3,GRIN2B,HTR3B,SLC1A1,SLC1A2,GABRA1,ABCB1,OPRM1,KCNH5
Neurotransmitter receptor activity involved in regulation of postsynaptic membrane potential	2.58E-09	GABRG2,GRIA2,GRIA3,GRIK2,GRIK3,GRIN2B,GABBR1,GABRA1
Cation channel activity	9.12E-09	DRD4,HTR1B,HTR3A,KCNH2,HTR3E,GRIA2,GRIA3,HTR3C,HTR3D,GRIK2,GRIK3,GRIN2B,HTR3B,OPRM1,KCNH5
Oxidoreductase activity, acting on paired donors, with incorporation or reduction of molecular oxygen	9.29E-09	TH,CYP2C19,CYP2D6,CYP2E1,CYP3A4,CYP3A5,ESR1,TPH2,AKT1,DBH,TPH1
Dopamine neurotransmitter receptor activity	3.34E-08	DRD1,DRD2,DRD3,DRD4
Glutamate receptor activity	3.39E-08	GRIA2,GRIA3,GRIK2,GRIK3,GRIN2B,GRM5
**GO: Cellular Components**		
Neuron projection	1.89E-38	SLC6A3,SLC6A4,GABRG2,GAD1,GAD2,TH,DRD1,DRD2,HTR1A,DRD4,HTR1B,HTR2A,HTR3A,COMT,NRCAM,NRXN1, HTR7,MAP2,SLC18A1,MTOR,SLC18A2,NTRK1,NTRK2,ESR1,NTRK3,ESR2,HTR3E,TPH2,CNTNAP2,ARC,SYP, GRIA2, GRIA3,DLG4,HTR3C,HTR3D,GRIK2,GRIK3,DBH,GRIN2B,HTR3B,CTNNB1,NR3C1,GRM5,FAS,CREB1, SLC1A1, SLC1A2, AR,HOMER1,CRH,BDNF,NPY,GABBR1,SOD1,GABRA1,OPRM1,TPH1
Synapse	6.03E-35	SLC6A3,SLC6A4,GABRG2,GAD1,GAD2,TH,DRD1,DRD2,DRD3,HTR1A,DRD4,HTR1B,NRXN3,HTR2A,HTR3A, COMT,NRCAM,NRXN1,HTR7,MAP2,SLC18A1,MTOR,SLC18A2,NTRK2,ESR1,NTRK3,HTR3E,CNTNAP2, ARC,SYP,GRIA2,GRIA3,DLG4,HTR3C,HTR3D,AKT1,GRIK2,GRIK3,DBH,GRIN2B,HTR3B,CTNNB1,NR3C1,GRM5, SLC1A1,SLC1A2,HOMER1,CRH,BDNF,NPY,GABBR1,NOS1AP,GABRA1,OPRM1
Somatodendritic compartment	1.10E-30	SLC6A3,GABRG2,TH,DRD1,DRD2,HTR1A,DRD4,HTR1B,HTR2A, HTR3A,COMT,NRXN1,HTR7,MAP2, MTOR,SLC18A2,NTRK1, NTRK2,ESR1,ESR2,CNTNAP2,ARC,GRIA2,GRIA3,DLG4,GRIK2, GRIK3,DBH,GRIN2B,HTR3B,CTNNB1,NR3C1,GRM5,FAS,SLC1A1,SLC1A2,AR,HOMER1,CRH,BDNF,NPY, GABBR1,SOD1,GABRA1,OPRM1
Neuronal cell body	1.85E-25	SLC6A3,TH,DRD1,DRD2,HTR1A,DRD4,HTR2A,HTR3A,NRXN1,HTR7, MAP2,MTOR,SLC18A2,NTRK1,NTRK2,ESR1, ESR2, CNTNAP2,ARC,GRIA2,GRIA3,GRIK2,GRIK3, DBH,GRIN2B,HTR3B,FAS,SLC1A1, HOMER1,CRH, BDNF,NPY,GABBR1,SOD1,OPRM1
Dendritic tree	2.07E-25	GABRG2,TH,DRD1,DRD2,HTR1A,DRD4,HTR1B,HTR2A,COMT,HTR7, MAP2,MTOR,NTRK1,NTRK2,CNTNAP2, ARC,GRIA2,GRIA3,DLG4, GRIK2,GRIK3,DBH,GRIN2B,CTNNB1,NR3C1,GRM5,FAS,SLC1A1, SLC1A2,AR,HOMER1,BDNF,GABBR1,SOD1,GABRA1,OPRM1
Intrinsic component of synaptic membrane	1.23E-24	SLC6A3,SLC6A4,GABRG2,DRD1,DRD2,DRD3,HTR1A,DRD4, HTR1B,HTR2A,HTR3A,NRCAM,NRXN1, HTR7,NTRK3,GRIA2,GRIA3,DLG4,GRIN2B,GRM5,SLC1A2,GABBR1,GABRA1,OPRM1
glutamatergic synapse	2.06E-18	GABRG2,DRD1,DRD2,DRD3,DRD4,HTR2A,HTR3A,NRCAM,NRXN1, MTOR,NTRK2,NTRK3,ARC,GRIA2,GRIA3, DLG4,GRIK2,GRIK3, GRIN2B,NR3C1,GRM5,SLC1A2,HOMER1,GABBR1,NOS1AP
Neuron spine	5.57E-18	DRD1,DRD2,DRD4,COMT,NTRK2,CNTNAP2,ARC,SYP,GRIA2, GRIA3,DLG4,GRIK2,GRIN2B, NR3C1,GRM5,SLC1A1,SLC1A2, HOMER1,GABBR1,OPRM1
Distal axon	2.31E-17	GAD1,TH,DRD1,DRD2,DRD4,HTR1B,NRXN1,HTR7,MAP2,SLC18A1, SLC18A2,NTRK2,ESR1,SYP, GRIA2,GRIA3,GRIK2,GRIK3, DBH,GRIN2B,SLC1A1,BDNF,NPY
Terminal bouton	4.14E-16	TH,DRD4,SLC18A1,SLC18A2,NTRK2,ESR1,SYP,GRIA2, GRIA3,GRIK2,GRIK3,DBH, GRIN2B,BDNF,NPY
Dendritic spine	1.68E-15	DRD1,DRD2,DRD4,COMT,NTRK2,ARC,GRIA2,GRIA3,DLG4,GRIK2,GRIN2B,NR3C1,GRM5,SLC1A1,SLC1A2, HOMER1,GABBR1,OPRM1
Dendritic shaft	1.46E-13	DRD1,HTR2A,MAP2,GRIA2,GRIA3,CTNNB1,GRM5,SLC1A1,SLC1A2,HOMER1,GABBR1
Perikaryon	2.04E-13	TH,DRD2,NTRK2,ESR1,ESR2,CNTNAP2,GRIA2,GRIA3,GRIK2, GRIK3,SLC1A1,CRH,BDNF,NPY,OPRM1
Asymmetric synapse	6.82E-12	DRD1,DRD2,DRD3,NRCAM,MAP2,NTRK2,ARC,SYP, GRIA2,GRIA3,DLG4,GRIK2,GRIN2B,CTNNB1, NR3C1,GRM5,SLC1A1,HOMER1
Neuron to neuron synapse	1.87E-11	DRD1,DRD2,DRD3,NRCAM,MAP2,NTRK2,ARC,SYP,GRIA2, GRIA3,DLG4,GRIK2,GRIN2B,CTNNB1, NR3C1,GRM5,SLC1A1,HOMER1
Receptor complex	3.58E-10	GABRG2,HTR1B,HTR2A,HTR3A,NTRK1,NTRK2,NTRK3,GRIA2,GRIA3,DLG4,GRIK2,GRIK3, GRIN2B,HTR3B, GDNF,IL6,GABBR1,GABRA1
GABA-ergic synapse	5.15E-10	GABRG2,DRD1,DRD2,DRD3,HTR1A,DRD4,NRXN3,NRXN1,GABBR1,GABRA1

### Predicted transcription factors and microRNAs for target genes

**Fig. 3:**
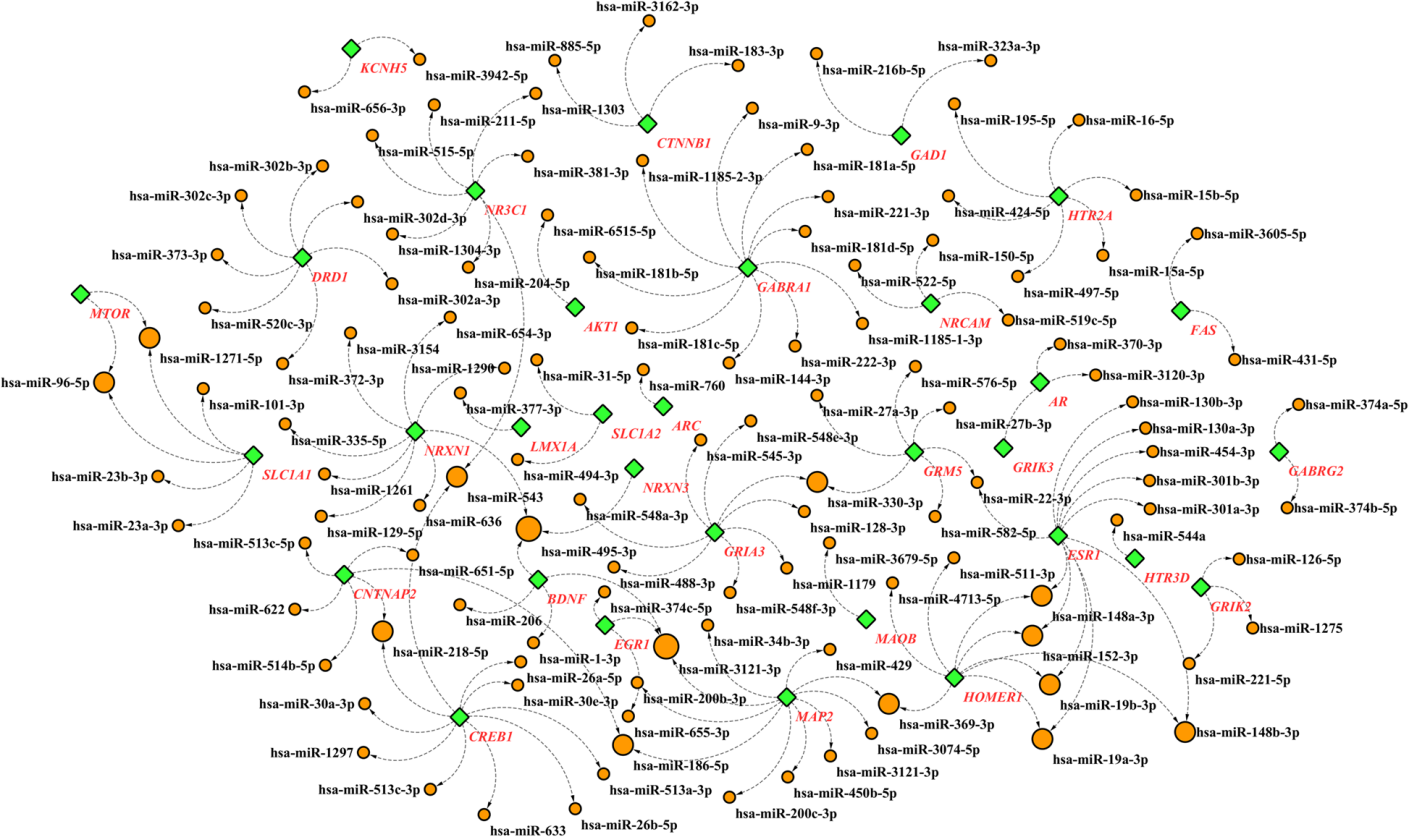
Gene-miRNAs interaction network for common genes between SCZ and OCD. In the current network each shared gene (green nodes) targeting some significant miRNAs (orange nodes). miRNAs with more degrees are represented larger than others

As regards gene expression patterns strongly regulate at transcriptional and posttranscriptional levels, the importance of the next steps becomes apparent. TF analysis predicted 15 significant TFs, namely HMGA1, MAPK14, HINFP, TEAD2, JUND, NFIA, SAMD9L, NFKB1, REL, PRDM1, HOXA5, GATA1, NKX3-1, VDR and STAT3 (Table.2). We also predicted 121 miRNAs with target score of more than 95 through miRDB database for our target genes (Fig. 3). Reconstructed gene-miRNAs interaction network showed that two miRNAs hsa-miR-3121-3p and hsa-miR-495-3p represented three interactions with their target genes meanwhile hsa-miR-369-3p, hsa-miR-186-5p, hsa-miR-218-5p, hsa-miR-22-3p, hsa-miR-330-3p, hsa-miR-543, hsa-miR-1271-5p, hsa-miR-96-5p, hsa-miR-148b-3p, hsa-miR-152-3p, hsa-miR-148a-3p, hsa-miR-19b-3p and hsa-miR-19a-3p interacted with two different targeted genes (Fig. 3). Furthermore, hsa-miR-144-3p, hsa-miR-22-3p, hsa-miR-221-3p, hsa-miR-3121-3p, hsa-miR-19a-3p, hsa-miR-200c-3p, hsa-miR-429, hsa-miR-381-3p, hsa-miR-126-5p, hsa-miR-200b-3p, hsa-miR-222-3p, and hsa-miR-488-3p had target prediction scores more than 99.

**Table 2:** Result of transcription factor prediction for SCZ-OCD shared genes

Name	P-value	Involved Genes
HMGA1	0.0001071	GRIA2, GABRA1, ABCB1, BDNF, NRXN1, GSTP1, NRXN3, HTR3D, HTR3A, PRL,OPRM1,NR3C1, RGS4, NOS1AP, UCP2,DRD2,GRIA3
MAPK14	0.0002271	GABRA1, KCNH5, SLC1A1, OPRM1, NR3C1, ESR1, RGS4,AR, NOS1AP,CRH, DRD1, ANKK1, SLC18A1, DRD3,SLC18A2
HINFP	0.0002323	CNTNAP2, MAOB, COMT, NR3C1, SLC6A3, RGS4, AKT1, NRCAM, DRD2,SLC18A2, KCNH2, NTRK1,NTRK2,EGR1,GABRA1,BDNF,NTRK3,OLIG2,OPRM1,HTR3B,ESR1,AR, ARC,NOS1AP,CRH,CYP2E1,HLA-DRB1
TEAD2	0.0007907	HOMER1, BDNF, NTRK3, GRIK3, HTR2A,COMT, NR3C1, SLC6A3, LMX1A,TH, GDNF, DLG4, UCP2, CTNNB1, ANKK1, SLC18A2,
JUND	0.001069	GRIA2, GABRA1, MAOB, BDNF, NRXN1, NRXN3, PRL,NR3C1, CYP3A5,GABRG2, HTR7, NPY, CRH, NRCAM, SLC18A1
NFIA	0.001384	MAOB, PRL,NR3C1, CYP3A4, ESR1, ESR2, SOD1, AR, GRM5, LMX1A, TH, CYP2D6, DLG4, CYP2E1, DRD3
SAMD9L	0.003779	KCNH2, NPSR1,TPH2,GRIA2,EGR1,MOG,HTR3D, GAD2, HTR3A,NR3C1,CYP3A5,ESR1,DRD1,DRD3
NFKB1	0.004332	GSTP1, SLC1A1,NRXN3,COMT, SLC6A3, SLC6A4, AKT1, EGR1, EGR2, KCNH5, BDNF, NTRK3, GAD1, GAD2, OPRM1, GRIN2B, ESR1, GABRG2, MTOR, ESR2,ARC,IL6, CREB1, GDNF, NOS1AP,ANKK1
REL	0.00493	KCNH5, LMX1A, BDNF, NTRK3,PRL,SLC6A4
PRDM1	0.005229	NTRK2, GABRA1, BDNF, NFKBIL1, NRXN1, NRXN3, DBH, OPRM1, COMT, NR3C1, RGS4, CREB1, GDNF, HLA-DRB1
HOXA5	0.005306	CNTNAP2, GRIA2, GABRA1, GABBR1, EGR2, MAOB, BDNF, SLC1A1, SLC1A2,NRXN3, HTR1A, HTR3D, GAD2, HTR3A, OLIG2, OPRM1, COMT, NR3C1, SLC6A3, GDNF, MAP2, NRCAM, DRD3
GATA1	0.006189	NPSR1, NTRK2, EGR1, ABCB1, BDNF, NRXN3, PRL, OPRM1, SYP, CYP3A4, ESR1, RGS4, GRM5, HTR7, MAP2, DLG4, NPY, CRH, FAS, DRD3, SLC18A2
NKX3-1	0.006509	KCNH5, NRXN3, ESR1, MTOR
VDR	0.007118	BDNF, CYP3A4, CYP3A5
STAT3	0.008629	GRIA2, TPH1, GABBR1, EGR2, ABCB1, MOG, BDNF, NFKBIL1, NTRK3, GAD1, DBH, OPRM1, CYP3A5, ESR1, SLC6A4, RGS4, AR, IL6, MAP2, NRCAM, HLA-DRB1

### Repurposed drug and gene-drug interaction network for SCZ-OCD associated genes

**Fig. 4:**
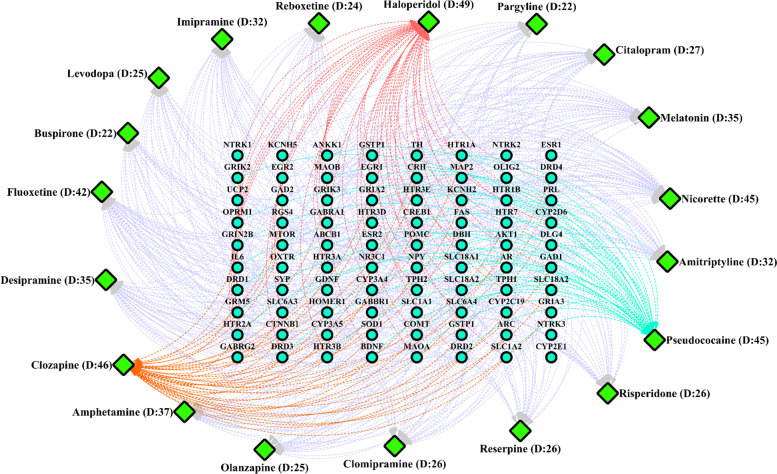
Gene-drug interaction network of SCZ-OCD shared genes. In the current network each shared genes (circle nodes) targeting some drugs (diamond nodes)."D" is considered as degree values for related drugs in the network

Based on results from ToppGene database, we repurposed 19 potential significant drugs, namely Haloperidol, Clozapine, Desipramine, Fluoxetine, Nicorette, Pseudococaine, Amitriptyline, Amphetamine, Risperidone, Clomipramine, Reboxetine, Imipramine, Reserpine, Citalopram, Levodopa, Pargyline, Melatonin, Olanzapine and Buspirone for SCZ-OCD-associated common genes (Fig. 4). In addition, the gene-drug interaction network revealed that four drugs Haloperidol, Clozapine, Fluoxetine and Melatonin were more connected drugs according to their degrees (Fig. 4).

## Discussion

Previous studies have shown that SCZ is closely related to OCD [13, 15–19]. In the present study, we used globally accessible databases (Geneweaver and Harmonizome) for exploiting related genes of SCZ and OCD to survey (by conducting bioinformatic analyses) the hypothesis that these two mental disorders display prospective significant shared genetic basis, potential biomarkers and therapeutic targets in terms of common genes. Initially, we found a big common gene set (with 89 genes) between SCZ and OCD which can be evidence for the common pathogenesis and co-occurrence of these two disorders. The results of enrichment analysis on the common genes showed that ten genes (*BDNF, SLC6A4, GAD1, HTR2A, GRIN2B, DRD2, SLC6A3, COMT, TH* and *DLG4*) are the most central genes in associated with both disorders.

*BDNF* was found as the most central shared gene (Fig. 1). This gene encodes BDNF protein which is a member of a large family of neuronal growth factors called neurotrophins and plays a pivotal role in neurogenesis, differentiation and cell survival [33]. Meanwhile, it contributes to the transcription and translation of proteins involved in the synaptogenesis, development and stability of synapses [34]. BDNF has been widely studied in psychiatric disorders and its possible roles in the pathophysiology of them were discussed in the last two decades. It is released at the synapse and affects synaptic plasticity, subsequently induces critical changes in cognitive functions, learning and memory [35, 36]. While, a defect in the regulation of BDNF release can cause abnormalities in the underlying brain processes and cognitive dysfunctions in the psychiatric disorders [37]. BDNF signaling may substantially promote the structure and functioning of several neural circuits involved in the modulation of various neurotransmitter systems, including the dopaminergic [38], serotoninergic [39] and GABAergic [40] systems, all closely related to SCZ. In this regard, the normal development of these systems may disturb by dysfunction of BDNF-TrkB signaling during critical developmental periods, consequently leading to physiological dysregulation and vulnerability to SCZ[41]. Relating to OCD, it has been demonstrated that sequence variants in the *BDNF* gene are strongly associated with OCD [42]. For example, it was reported that the Val66Met *BDNF* gene variant contributes to the OCD pathobiology [43, 44]. In addition, more studies have shown that this variant lead to functional differences and consequently decrease the activity of the BDNF system, which can be a risk factor for OCD [45].

Furthermore, the interface between SCZ and OCD can be explained through the serotoninergic neurotransmission as two important common genes are *SLC6A4* and *HTR2A* which have critical functions in the serotoninergic pathway. *SLC6A4* by the encoding of serotonin transporter (5-HTT) has a crucial role in the regulation of serotonin via reuptake of it from synaptic clefts [46]. Because this gene is involved in the pathogenesis of SCZ, it is considered as a candidate gene for SCZ [47]. It has been reported that mRNA and protein levels of 5-HTT were changed in SCZ patients compared with healthy subjects [48, 49]. Likewise, there is a lot of evidence reporting that serotonergic system is implicated in the pathophysiology of OCD, in particular well-known anti-obsessional efficacy of selective serotonin reuptake inhibitors (SSRIs) have suggested an important role for 5-HTT in the etiology of OCD [50]. Zitterl et al. showed a significant reduction in 5-HTT availability in the brain regions of OCD patients [51]. Clearly, several studies demonstrated that there is a significant association between the *SLC6A4* polymorphisms and OCD susceptibility [52]. The *HTR2A* gene encodes the serotonin receptor type 2A (5-HTR2A) and is abundantly expressed in the glutamatergic neurons and GABA-ergic interneurons in the prefrontal cortex and hippocampal regions which both neurotransmission systems are well known to be associated with SCZ [53]. It has been shown that 5-HTR2A is involved in the pathogenesis of SCZ as its activation regulates both dopaminergic and glutamatergic transmission in the brain [54]. Relating to OCD, findings from candidate gene studies demonstrated that *HTR2A* is a most important gene for development of the disorder [55]. Also, recent meta-analytic evidence showed that polymorphic variants within this gene are significantly associated with OCD pathogenesis [56, 57].

*GAD1* is one of the other important genes in both SCZ and OCD. It encodes the glutamic acid decarboxylase-67 (GAD67) enzyme in multiple cortical regions (particularly in the prefrontal cortex and hippocampus) which is responsible for most cortical γ-Aminobutyric acid (GABA) synthesis [58]. This gene with a lower expression level in the SCZ subjects and consequently lower levels of GAD67 mRNA and protein is a well-known biomarker for SCZ [59]. In OCD patients, a recent clinical study reported that GABA abnormalities can be found within the anterior cingulate cortex [60]. Besides, Zhang et al. indicated that GABA concentration in the prefrontal cortex contributes to the psychopathology of OCD [61].

According to our results, another candidate gene for co-occurrence of SCZ and OCD is *GRIN2B* encodes the NR2B subunit of N-methyl D-aspartate (NMDA) glutamate receptors and may play an important role in synaptic plasticity, circuit formation and brain development [62]. Recently, it has been reported that variations in *GRIN2B* can be associated with SCZ which may be due to abnormalities of the NR2B subunit and consequently altered function of NMDA receptors [63]. In addition, hypofunction of NMDA receptors has been suggested as a mechanism in the pathogenesis of SCZ, based on the point that noncompetitive antagonists of the NMDA receptors, such as ketamine and phencyclidine, induce SCZ-like symptoms [64]. In contrast, it is proposed that neuronal excitotoxicity resulting from hyperactive NMDA receptors has an important role in SCZ [65]. Clinical and preclinical evidence have suggested that dysregulation of glutamatergic system contributes to the etiology of OCD [66]. In imaging studies of OCD, it has been shown that a hyperglutamatergic dysfunction may lead to abnormalities in the cortico–striatal–thalamo–cortical circuits [67]. In addition, it is demonstrated that the *GRIN2B* gene is implicated in OCD as mutations of it have been associated with the disorder in males [68] and the presence of contamination obsessions and cleaning compulsions [69].

The *DRD2* gene, coding for dopamine D2 receptor, is an attractive candidate gene for SCZ due to its role in dopaminergic system [70]. Several polymorphisms of this gene have been identified related to SCZ, hence it is well considered as a causative factor in SCZ [71]. Besides, *DRD2* likely contributes to the OCD pathology since it has been demonstrated that the dopaminergic system is implicated in inducing or aggravating the symptoms of OCD [72] and in particular it is reported that the *DRD2* A2 allele is significantly higher in male OCD patients compared to controls[73].

Other important shared genes between SCZ and OCD are *SLC6A3, COMT, TH* and *DLG4*. The *SLC6A3* gene encodes the dopamine transporter and can be determinative in the regulation of dopamine in the synaptic cleft. It has been well indicated that its polymorphisms are risk factors for SCZ [74]. Also, the manifestation of OCD symptoms can be associated with *SLC6A3* [75]. Since the COMT enzyme has a crucial role in the metabolism of dopamine, the *COMT* gene is considered as an important factor in the etiology of SCZ [76]. Higher dopamine levels are likely involved in OCD, hence *COMT* can be a suitable candidate for OCD [77]. Tyrosine hydroxylase (TH), encoded by *TH* gene, is a rate-limiting enzyme that produces dopamine in the brain and can be related to SCZ [78]. Dopamine neurotransmission is likely associated with OCD [77]. Therefore, the TH gene may be involved in OCD. Post-synaptic density protein 95 (PSD95), encoded by the *DLG4* gene, has an essential rolein regulating NMDA receptor activity and altered expression of DLG4 has been revealed in the post-mortem brain of subjects with SCZ [79]. In OCD pathogenesis, since glutamatergic system can be disrupted [80], *DLG4* may be an interference factor in this system.

In our study, the significant molecular functions exhibit receptor activity as the most common of them associated with SCZ and OCD. As recently reviewed, multiple serotonin receptors (5-HTR) are implicated in SCZ, including 5-HT1AR, 5-HT2CR, 5-HT3R, 5-HT6R, 5-HT7R and several studies link 5-HT2AR to the pathogenesis of SCZ as its activation regulates both dopaminergic and glutamatergic transmission [54]. It is worth mentioning in SCZ that the more-focused and well-known receptors are dopamine D2 receptors involved in the mesolimbic dopamine pathway, and their hyperactivity is responsible for the cause of the positive symptoms of SCZ, with all antipsychotics acting to block them [81]. Also, Dopamine D2 receptors likely play a part in the OCD pathology as decreased striatal D2/D3 and D1 receptors were reported in patients with OCD [82]. Positron emission tomography (PET) showed a significant reduction of 5-HT2AR availability in OCD patients compared to healthy subjects [83].

Based on cellular component enrichment analysis, the cellular components involved in the transition of the signals, namely, neuron projection and synapse were the most important cellular components in both SCZ and OCD. As mentioned, serotonergic synapse markers (5-HTT and 5-HT2AR) contributed to the development of OCD. Besides, the neurocircuitry of OCD is located between different brain regions which are densely innervated by neuron projections of the serotonergic and dopaminergic neurotransmitter systems [84]. Also, Rosoklija et al. reported the structural abnormalities of dendrites in SCZ and mood disorders [85]. In addition, Faludi and Mirnics showed that synaptic disturbances are important and integral part of SCZ pathophysiology [86].

KEGG pathway analysis indicated that the identified common genes mostly contributed to serotonergic synapse, cocaine addiction, dopaminergic synapse, amphetamine addiction, alcoholism, taste transduction, glutamatergic synapse, tyrosine metabolism and estrogen signaling pathways. As discussed above, several studies have shown that serotonergic pathway has a substantial role in the pathophysiology of SCZ and OCD. In addition to the dopamine and glutamate theory of psychosis, there is also the serotonin theory states that cortical 5-HT2AR hyperfunction can also result in psychosis [81].

TF analysis predicted that HMGA1, MAPK14, HINFP and TEAD2 are the more relevant TF biomarkers associated with both SCZ and OCD. According to the gene cards database (www.genecards.org); HMGA1 is associated with Type 2 Diabetes Mellitus and Multiple Lipomatosis. Likewise, Diseases associated with MAPK14 include Chlamydia and Alexander Disease. HINFP show an association with Cerebro-oculo-facio-skeletal Syndrome 2 and Fetal Alcohol Spectrum Disorder. Diseases associated with TEAD2 include Sveinsson Chorioretinal Atrophy and Multiple Acyl-CoA Dehydrogenase Deficiency [87].

This study predicted 121 significant miRNAs for common genes through miRDB database (Fig. 3). Among them, hsa-miR-3121-3p, hsa-miR-495-3p, hsa-miR-369-3p, hsa-miR-186-5p, hsa-miR-218-5p, hsa-miR-22-3p, hsa-miR-330-3p, hsa-miR-543, hsa-miR-1271-5p, hsa-miR-96-5p, hsa-miR-148b-3p, hsa-miR-152-3p, hsa-miR-148a-3p, hsa-miR-19b-3p and hsa-miR-19a-3p were more relevant identified biomarkers in associated to both SCZ and OCD. In human, almost 70% of miRNAs express in the nervous system [88] and they have a role in the regulation of neural structure and function such as formation of dendrites and dendritic spines, axon growth, neural developments and the maturation process [89]. Also, abnormal expressions of miRNAs could be important as they are involved in the occurrence of neuropsychiatric disorders [90]. Therefore, considering them as potential biomarkers for co-occurrence of SCZ and OCD can be useful for more diagnostic and therapeutic targets.

A class of non-coding RNA molecules as miRNAs act as negative regulators of post-transcriptional processes that mediate gene expression profile and subsequent biological functions. In the present study, we predicted 121 miRNAs with target predicting score of more than 95 concerning SCZ and OCD. Pan et al., showed that hsa-miR-144-3p is associated with schizophrenia through a decrease in the expression of ATPase Na^+^/K^+^ transporting subunit beta 2 (ATP1B2) and PI3K/Akt/mTOR signalling in rat hippocampus [91]. It has been reported that miR-3121-3p suppressed RAP1 GTPase activating protein (Rap1GAP) [92], while this protein is crucial for development of dentate gyrus and prevention of SCZ-like behavior in mice [93]. Importantly next-generation sequencing and real-time quantitative polymerase chain reaction (qRT-PCR) on peripheral blood cells in patients with SCZ, suggested hsa-miR-22-3p as a biomarker for these patients [94]. Interestingly, the level of hsa-miR-22-3p also significantly increased in patients with OCD compared to healthy subjects [95]. A bioinformatics study on microarray dataset from brain post-mortem samples of SCZ patients identified hsa-miR-26a-5p as a differentially expressed miRNA compared to control samples [96]. Also, comparison of expression pattern of several miRNAs in peripheral blood cells by qRT-PCR revealed a significant increase in the expression of miR-26a-5p in OCD patients compared to control subjects [97].

Finally, current research identified drugs (particularly Haloperidol, Fluoxetine, Clozapine and Melatonin) that may have a potential influence on the co-occurrence of SCZ and OCD. Haloperidol, a highly effective first-generation antipsychotic (FGA), is one of the most used antipsychotic drugs, and it has very high anti-dopaminergic activity in the mesolimbic dopamine pathway [98] hence it is very efficient for the treatment of SCZ [99]. However, like other FGAs, it is associated with severe extrapyramidal side effects [100]. Haloperidol is considered as second in line with antipsychotic augmenting agents in OCD patients who showed an inadequate response to SSRIs but better response with antipsychotic augmentation [101]. Fluoxetine is one of the oldest SSRIs and is applied as a first-line drug for the treatment of some mental illnesses such as major depressive disorder, premenstrual dysphoric disorder, panic disorder and bulimia nervosa [102]. Furthermore, it is widely used in OCD patients due to its good therapeutic response, good compliance and low side effects [103, 104]. Also, in treating SCZ, it has been indicated that the use of antidepressants like fluoxetine as an add-on therapy to antipsychotics can improve the negative symptoms in patients with chronic SCZ [105]. Clozapine, a dibenzodiazepine developed in 1961, is an antipsychotic approved in treating resistant SCZ [106]. It has been indicated that clozapine is more effective than any other antipsychotic drug (first or second-generation) in the treatment of resistant SCZ [107]. However, the anti-serotonergic effects of clozapine in cortico-striatal serotoninergic circuits may induce OCD-like behavior in mice and generate OCS in patients treated with it [108, 109]. Therefore, it is hypothesized that decrease plasma concentration of clozapine may alleviate OCD, but it can exacerbate the severity of SCZ [20]. Clinicians should be aware of the exacerbation of OCD and OCS after chronic prescription of clozapine in treatment of SCZ. However, prescription of SSRIs along with antipsychotic medications such as clozapine and olanzapine might be a proposed alternative treatment in co-occurrence of SCZ and OCD. As, Stryjer et al. reported that administration of 20 mg/day escitalopram in patients with SCZ and OCD that were treated with antipsychotic drugs (i.e. clozapine, risperidone and quetiapine), decreased the total Yale Brown Obsessive–Compulsive Scale scores (Y-BOCS, *P* = 0.001) and Positive and Negative Syndrome Scale scores (PANSS, *P* = 0.03) [110]. Melatonin, the endogenous hormone that regulates circadian rhythms, is used exogenously for the treatment of sleep disorders [111]. Available evidence has suggested that melatonin is linked to SCZ. Sleep disorder is a usual feature of SCZ [112] and appear to be caused by abnormal melatonin functions and abnormal circadian implicated in the pathophysiology of SCZ [113]. Additionally, decreased melatonin level in SCZ patients was reported [114–116]. A recent systematic review of melatonin use for SCZ showed that adjunctive melatonin therapy can be useful for sleep, metabolic profile and tardive dyskinesia in SCZ patients [117]. Sleep disorders are also prevalent among OCD patients, as up to 48% of them report these disorders [118]. Furthermore, Monteleone et al. showed that overall plasma concentrations of melatonin are lower in patients with OCD compared with normal controls [119]. Hence, melatonin therapy may be helpful for OCD. Our study utilized a bioinformatic method to identify common molecular and cellular mechanisms and predict new therapeutic drugs for SCZ and OCD. However, the present study has a number of limitations. One limitation was the present study failed to verify main findings with experimental results due to lack of confirmatory experimental animal studies that mimic SCZ and OCD-like behavior. Another limitation is lack of an integrated comprehensive database to support genetic basis of OCD in human subjects. Furthermore, the function of most predicted miRNAs have yet to be determined. Therefore, validation of predicted results requires further in vitro, in vivo, and especially clinical future experimental researches with large sample size.

## Conclusion

Obsessive–compulsive behavior is a common comorbid condition with schizophrenia. Herein, we conducted a comprehensive enrichment analysis on the common genetic basis of SCZ and OCD. Regarding comorbid disorders, bioinformatics studies may help us to better understand the common pathophysiology of these disorders by identifying possible biomarkers and underlying mechanisms involved in them. Furthermore, finding more potential therapeutic options for disorders can be another implication of such analyses and future experimental studies.

## Supplementary Information

**Additional file 1.**

## Data Availability

All data sets generated/analyzed for this study are included in the manuscript.
