# Supplementary material for: Disclosing common biological signatures and predicting new therapeutic targets in schizophrenia and obsessive–compulsive disorder by integrated bioinformatics analysis
Source: BMC Psychiatry. 2023 Jan 14;23:40. doi: 10.1186/s12888-023-04543-z (PMC9840830; doi:10.1186/s12888-023-04543-z)
Supplement: Supplementary file 1 — Additional file 1. [file 12888_2023_4543_MOESM1_ESM.docx]

**Disclosing** **common biological signatures and predicting new therapeutic targets in schizophrenia and obsessive-compulsive disorder by i****ntegrated bioinformatics analysis**

**Supplementary Tables**

| Supplementary.Table.1 All genes associated with Schizophrenia extracted from GeneWeaver and Harmonizome. | | |
| --- | --- | --- |
| Index | **Genes symbol** | **Gene full name** |
|  | ABAT | 4-aminobutyrate aminotransferase |
|  | ABCA1 | ATP-binding cassette, sub-family A (ABC1), member 1 |
|  | ABCA7 | ATP-binding cassette, sub-family A (ABC1), member 7 |
|  | ABCB1 | ATP-binding cassette, sub-family B (MDR/TAP), member 1 |
|  | ACE | angiotensin I converting enzyme |
|  | ACLY | ATP citrate lyase |
|  | ACSL6 | acyl-CoA synthetase long-chain family member 6 |
|  | ACSM1 | acyl-CoA synthetase medium-chain family member 1 |
|  | ACVR1B | activin A receptor, type IB |
|  | ADAM15 | ADAM metallopeptidase domain 15 |
|  | ADAM22 | ADAM metallopeptidase domain 22 |
|  | ADAM28 | ADAM metallopeptidase domain 28 |
|  | ADAMTS4 | ADAM metallopeptidase with thrombospondin type 1 motif, 4 |
|  | ADCYAP1 | adenylate cyclase activating polypeptide 1 (pituitary) |
|  | ADIPOQ | adiponectin, C1Q and collagen domain containing |
|  | ADORA1 | adenosine A1 receptor |
|  | ADORA2A | adenosine A2a receptor |
|  | ADRA1A | adrenoceptor alpha 1A |
|  | ADRA2A | adrenoceptor alpha 2A |
|  | ADRA2C | adrenoceptor alpha 2C |
|  | ADRB3 | adrenoceptor beta 3 |
|  | ADSS | adenylosuccinate synthase |
|  | AGAP1 | ArfGAP with GTPase domain, ankyrin repeat and PH domain 1 |
|  | AGBL1 | ATP/GTP binding protein-like 1 |
|  | AHI1 | Abelson helper integration site 1 |
|  | AJAP1 | adherens junctions associated protein 1 |
|  | AKT1 | v-akt murine thymoma viral oncogene homolog 1 |
|  | AKT2 | v-akt murine thymoma viral oncogene homolog 2 |
|  | ALDH1A1 | aldehyde dehydrogenase 1 family, member A1 |
|  | ALDH1A2 | aldehyde dehydrogenase 1 family, member A2 |
|  | ALDH1A3 | aldehyde dehydrogenase 1 family, member A3 |
|  | ALDH3A1 | aldehyde dehydrogenase 3 family, member A1 |
|  | ALDH3B1 | aldehyde dehydrogenase 3 family, member B1 |
|  | ALDH5A1 | aldehyde dehydrogenase 5 family, member A1 |
|  | AMT | aminomethyltransferase |
|  | ANK3 | ankyrin 3, node of Ranvier (ankyrin G) |
|  | ANKK1 | ankyrin repeat and kinase domain containing 1 |
|  | ANKRD11 | ankyrin repeat domain 11 |
|  | ANO3 | anoctamin 3 |
|  | AP3B1 | adaptor-related protein complex 3, beta 1 subunit |
|  | AP3B2 | adaptor-related protein complex 3, beta 2 subunit |
|  | AP3D1 | adaptor-related protein complex 3, delta 1 subunit |
|  | AP3M1 | adaptor-related protein complex 3, mu 1 subunit |
|  | AP3M2 | adaptor-related protein complex 3, mu 2 subunit |
|  | APBA2 | amyloid beta (A4) precursor protein-binding, family A, member 2 |
|  | APOB | apolipoprotein B |
|  | APOE | apolipoprotein E |
|  | APOL1 | apolipoprotein L, 1 |
|  | APOL2 | apolipoprotein L, 2 |
|  | APOL4 | apolipoprotein L, 4 |
|  | APOL5 | apolipoprotein L, 5 |
|  | APOL6 | apolipoprotein L, 6 |
|  | AQP4 | aquaporin 4 |
|  | AR | androgen receptor |
|  | ARHGAP18 | Rho GTPase activating protein 18 |
|  | ARHGEF10 | Rho guanine nucleotide exchange factor (GEF) 10 |
|  | ARID4B | AT rich interactive domain 4B (RBP1-like) |
|  | ARRB2 | arrestin, beta 2 |
|  | ARSG | arylsulfatase G |
|  | ARVCF | armadillo repeat gene deleted in velocardiofacial syndrome |
|  | ASPM | asp (abnormal spindle) homolog, microcephaly associated (Drosophila) |
|  | ASTN1 | astrotactin 1 |
|  | ASTN2 | astrotactin 2 |
|  | ATF2 | activating transcription factor 2 |
|  | ATF4 | activating transcription factor 4 |
|  | ATF5 | activating transcription factor 5 |
|  | ATM | ATM serine/threonine kinase |
|  | ATN1 | atrophin 1 |
|  | ATP2B2 | ATPase, Ca++ transporting, plasma membrane 2 |
|  | ATXN1 | ataxin 1 |
|  | ATXN3 | ataxin 3 |
|  | ATXN8OS | ATXN8 opposite strand (non-protein coding) |
|  | BAG3 | BCL2-associated athanogene 3 |
|  | BAP1 | BRCA1 associated protein-1 (ubiquitin carboxy-terminal hydrolase) |
|  | BDNF | brain-derived neurotrophic factor |
|  | BMP6 | bone morphogenetic protein 6 |
|  | BRD1 | bromodomain containing 1 |
|  | BSN | bassoon presynaptic cytomatrix protein |
|  | BTAF1 | BTAF1 RNA polymerase II, B-TFIID transcription factor-associated, 170kDa |
|  | BTLA | B and T lymphocyte associated |
|  | BTN2A2 | butyrophilin, subfamily 2, member A2 |
|  | BTN3A1 | butyrophilin, subfamily 3, member A1 |
|  | BTN3A2 | butyrophilin, subfamily 3, member A2 |
|  | CABIN1 | calcineurin binding protein 1 |
|  | CACNA1A | calcium channel, voltage-dependent, P/Q type, alpha 1A subunit |
|  | CACNA1B | calcium channel, voltage-dependent, N type, alpha 1B subunit |
|  | CACNA1C | calcium channel, voltage-dependent, L type, alpha 1C subunit |
|  | CACNG2 | calcium channel, voltage-dependent, gamma subunit 2 |
|  | CAD | carbamoyl-phosphate synthetase 2, aspartate transcarbamylase, and dihydroorotase |
|  | CALN1 | calneuron 1 |
|  | CAMKV | CaM kinase-like vesicle-associated |
|  | CAPN10 | calpain 10 |
|  | CAPN14 | calpain 14 |
|  | CARTPT | CART prepropeptide |
|  | CBS | cystathionine-beta-synthase |
|  | CBX2 | chromobox homolog 2 |
|  | CCDC13 | coiled-coil domain containing 13 |
|  | CCDC60 | coiled-coil domain containing 60 |
|  | CCK | cholecystokinin |
|  | CCKAR | cholecystokinin A receptor |
|  | CCL2 | chemokine (C-C motif) ligand 2 |
|  | CCR5 | chemokine (C-C motif) receptor 5 (gene/pseudogene) |
|  | CCSER1 | coiled-coil serine-rich protein 1 |
|  | CD48 | CD48 molecule |
|  | CDC25A | cell division cycle 25A |
|  | CDC42SE2 | CDC42 small effector 2 |
|  | CDH13 | cadherin 13 |
|  | CDKN2A | cyclin-dependent kinase inhibitor 2A |
|  | CERCAM | cerebral endothelial cell adhesion molecule |
|  | CERKL | ceramide kinase-like |
|  | CHGB | chromogranin B |
|  | CHI3L1 | chitinase 3-like 1 (cartilage glycoprotein-39) |
|  | CHL1 | cell adhesion molecule L1-like |
|  | CHRFAM7A | CHRNA7 (cholinergic receptor, nicotinic, alpha 7, exons 5-10) and FAM7A (family with sequence similarity 7A, exons A-E) fusion |
|  | CHRM1 | cholinergic receptor, muscarinic 1 |
|  | CHRM2 | cholinergic receptor, muscarinic 2 |
|  | CHRM5 | cholinergic receptor, muscarinic 5 |
|  | CHRNA3 | cholinergic receptor, nicotinic, alpha 3 (neuronal) |
|  | CHRNA4 | cholinergic receptor, nicotinic, alpha 4 (neuronal) |
|  | CHRNA5 | cholinergic receptor, nicotinic, alpha 5 (neuronal) |
|  | CHRNA7 | cholinergic receptor, nicotinic, alpha 7 (neuronal) |
|  | CHRNB2 | cholinergic receptor, nicotinic, beta 2 (neuronal) |
|  | CLDN5 | claudin 5 |
|  | CLINT1 | clathrin interactor 1 |
|  | CLOCK | clock circadian regulator |
|  | CLSTN2 | calsyntenin 2 |
|  | CLU | clusterin |
|  | CNNM2 | cyclin and CBS domain divalent metal cation transport mediator 2 |
|  | CNP | 2',3'-cyclic nucleotide 3' phosphodiesterase |
|  | CNR1 | cannabinoid receptor 1 (brain) |
|  | CNR2 | cannabinoid receptor 2 (macrophage) |
|  | CNTF | ciliary neurotrophic factor |
|  | COL7A1 | collagen, type VII, alpha 1 |
|  | COMT | catechol-O-methyltransferase |
|  | CPLX2 | complexin 2 |
|  | CPS1 | carbamoyl-phosphate synthase 1, mitochondrial |
|  | CRH | corticotropin releasing hormone |
|  | CRHBP | corticotropin releasing hormone binding protein |
|  | CRHR1 | corticotropin releasing hormone receptor 1 |
|  | CRHR2 | corticotropin releasing hormone receptor 2 |
|  | CSF2RA | colony stimulating factor 2 receptor, alpha, low-affinity (granulocyte-macrophage) |
|  | CSF2RB | colony stimulating factor 2 receptor, beta, low-affinity (granulocyte-macrophage) |
|  | CSMD1 | CUB and Sushi multiple domains 1 |
|  | CSPG5 | chondroitin sulfate proteoglycan 5 (neuroglycan C) |
|  | CTLA4 | cytotoxic T-lymphocyte-associated protein 4 |
|  | CTNND2 | catenin (cadherin-associated protein), delta 2 |
|  | CTXN3 | cortexin 3 |
|  | CYP1A2 | cytochrome P450, family 1, subfamily A, polypeptide 2 |
|  | CYP26A1 | cytochrome P450, family 26, subfamily A, polypeptide 1 |
|  | CYP26B1 | cytochrome P450, family 26, subfamily B, polypeptide 1 |
|  | CYP26C1 | cytochrome P450, family 26, subfamily C, polypeptide 1 |
|  | CYP2C19 | cytochrome P450, family 2, subfamily C, polypeptide 19 |
|  | CYP2D6 | cytochrome P450, family 2, subfamily D, polypeptide 6 |
|  | CYP2E1 | cytochrome P450, family 2, subfamily E, polypeptide 1 |
|  | CYP3A4 | cytochrome P450, family 3, subfamily A, polypeptide 4 |
|  | CYP3A5 | cytochrome P450, family 3, subfamily A, polypeptide 5 |
|  | CYP7B1 | cytochrome P450, family 7, subfamily B, polypeptide 1 |
|  | DAG1 | dystroglycan 1 (dystrophin-associated glycoprotein 1) |
|  | DAO | D-amino-acid oxidase |
|  | DAOA | D-amino acid oxidase activator |
|  | DBH | dopamine beta-hydroxylase (dopamine beta-monooxygenase) |
|  | DCC | DCC netrin 1 receptor |
|  | DCDC2 | doublecortin domain containing 2 |
|  | DDC | dopa decarboxylase (aromatic L-amino acid decarboxylase) |
|  | DDX31 | DEAD (Asp-Glu-Ala-Asp) box polypeptide 31 |
|  | DGCR2 | DiGeorge syndrome critical region gene 2 |
|  | DHFR | dihydrofolate reductase |
|  | DISC1 | disrupted in schizophrenia 1 |
|  | DLG1 | discs, large homolog 1 (Drosophila) |
|  | DLG4 | discs, large homolog 4 (Drosophila) |
|  | DLX1 | distal-less homeobox 1 |
|  | DMPK | dystrophia myotonica-protein kinase |
|  | DNMT3B | DNA (cytosine-5-)-methyltransferase 3 beta |
|  | DOC2A | double C2-like domains, alpha |
|  | DPYSL2 | dihydropyrimidinase-like 2 |
|  | DRD1 | dopamine receptor D1 |
|  | DRD2 | dopamine receptor D2 |
|  | DRD3 | dopamine receptor D3 |
|  | DRD4 | dopamine receptor D4 |
|  | DRD5 | dopamine receptor D5 |
|  | DRP2 | dystrophin related protein 2 |
|  | DTNBP1 | dystrobrevin binding protein 1 |
|  | DYM | dymeclin |
|  | EDIL3 | EGF-like repeats and discoidin I-like domains 3 |
|  | EFNB2 | ephrin-B2 |
|  | EGF | epidermal growth factor |
|  | EGR1 | early growth response 1 |
|  | EGR2 | early growth response 2 |
|  | EGR3 | early growth response 3 |
|  | EGR4 | early growth response 4 |
|  | EIF3D | eukaryotic translation initiation factor 3, subunit D |
|  | ELP3 | elongator acetyltransferase complex subunit 3 |
|  | EMC2 | ER membrane protein complex subunit 2 |
|  | ENO2 | enolase 2 (gamma, neuronal) |
|  | ENPP1 | ectonucleotide pyrophosphatase/phosphodiesterase 1 |
|  | ENTPD4 | ectonucleoside triphosphate diphosphohydrolase 4 |
|  | EPHA6 | EPH receptor A6 |
|  | ERBB3 | erb-b2 receptor tyrosine kinase 3 |
|  | ERBB4 | erb-b2 receptor tyrosine kinase 4 |
|  | ESR1 | estrogen receptor 1 |
|  | ESR2 | estrogen receptor 2 (ER beta) |
|  | FAAH | fatty acid amide hydrolase |
|  | FAM69A | family with sequence similarity 69, member A |
|  | FASN | fatty acid synthase |
|  | FBP1 | fructose-1,6-bisphosphatase 1 |
|  | FBXL21 | F-box and leucine-rich repeat protein 21 (gene/pseudogene) |
|  | FCRL3 | Fc receptor-like 3 |
|  | FEZ1 | fasciculation and elongation protein zeta 1 (zygin I) |
|  | FGF1 | fibroblast growth factor 1 (acidic) |
|  | FGFR1 | fibroblast growth factor receptor 1 |
|  | FGFR2 | fibroblast growth factor receptor 2 |
|  | FHIT | fragile histidine triad |
|  | FIGN | fidgetin |
|  | FLNB | filamin B, beta |
|  | FMO3 | flavin containing monooxygenase 3 |
|  | FNIP1 | folliculin interacting protein 1 |
|  | FOXP2 | forkhead box P2 |
|  | FRAXA | fragile site, folic acid type, rare, fra(X)(q27.3) A (macroorchidism, mental retardation) |
|  | FSTL1 | follistatin-like 1 |
|  | FXN | frataxin |
|  | FXR1 | fragile X mental retardation, autosomal homolog 1 |
|  | FXYD6 | FXYD domain containing ion transport regulator 6 |
|  | FZD3 | frizzled class receptor 3 |
|  | GABBR1 | gamma-aminobutyric acid (GABA) B receptor, 1 |
|  | GABRA1 | gamma-aminobutyric acid (GABA) A receptor, alpha 1 |
|  | GABRA5 | gamma-aminobutyric acid (GABA) A receptor, alpha 5 |
|  | GABRA6 | gamma-aminobutyric acid (GABA) A receptor, alpha 6 |
|  | GABRB2 | gamma-aminobutyric acid (GABA) A receptor, beta 2 |
|  | GABRG2 | gamma-aminobutyric acid (GABA) A receptor, gamma 2 |
|  | GABRP | gamma-aminobutyric acid (GABA) A receptor, pi |
|  | GAD1 | glutamate decarboxylase 1 (brain, 67kDa) |
|  | GAD2 | glutamate decarboxylase 2 (pancreatic islets and brain, 65kDa) |
|  | GAS2L1 | growth arrest-specific 2 like 1 |
|  | GCLC | glutamate-cysteine ligase, catalytic subunit |
|  | GCLM | glutamate-cysteine ligase, modifier subunit |
|  | GDNF | glial cell derived neurotrophic factor |
|  | GFRA2 | GDNF family receptor alpha 2 |
|  | GHRL | ghrelin/obestatin prepropeptide |
|  | GJA1 | gap junction protein, alpha 1, 43kDa |
|  | GLS | glutaminase |
|  | GLUD1 | glutamate dehydrogenase 1 |
|  | GLUL | glutamate-ammonia ligase |
|  | GMPS | guanine monphosphate synthase |
|  | GNAI2 | guanine nucleotide binding protein (G protein), alpha inhibiting activity polypeptide 2 |
|  | GNB1L | guanine nucleotide binding protein (G protein), beta polypeptide 1-like |
|  | GNB3 | guanine nucleotide binding protein (G protein), beta polypeptide 3 |
|  | GNL3 | guanine nucleotide binding protein-like 3 (nucleolar) |
|  | GPC1 | glypican 1 |
|  | GPM6A | glycoprotein M6A |
|  | GPX1 | glutathione peroxidase 1 |
|  | GRIA2 | glutamate receptor, ionotropic, AMPA 2 |
|  | GRIA3 | glutamate receptor, ionotropic, AMPA 3 |
|  | GRIA4 | glutamate receptor, ionotropic, AMPA 4 |
|  | GRID1 | glutamate receptor, ionotropic, delta 1 |
|  | GRIK3 | glutamate receptor, ionotropic, kainate 3 |
|  | GRIK4 | glutamate receptor, ionotropic, kainate 4 |
|  | GRIK5 | glutamate receptor, ionotropic, kainate 5 |
|  | GRIN1 | glutamate receptor, ionotropic, N-methyl D-aspartate 1 |
|  | GRIN2A | glutamate receptor, ionotropic, N-methyl D-aspartate 2A |
|  | GRIN2B | glutamate receptor, ionotropic, N-methyl D-aspartate 2B |
|  | GRIN2D | glutamate receptor, ionotropic, N-methyl D-aspartate 2D |
|  | GRIN3A | glutamate receptor, ionotropic, N-methyl-D-aspartate 3A |
|  | GRM2 | glutamate receptor, metabotropic 2 |
|  | GRM3 | glutamate receptor, metabotropic 3 |
|  | GRM4 | glutamate receptor, metabotropic 4 |
|  | GRM5 | glutamate receptor, metabotropic 5 |
|  | GRM7 | glutamate receptor, metabotropic 7 |
|  | GRM8 | glutamate receptor, metabotropic 8 |
|  | GSK3A | glycogen synthase kinase 3 alpha |
|  | GSK3B | glycogen synthase kinase 3 beta |
|  | GSTM1 | glutathione S-transferase mu 1 |
|  | GSTO1 | glutathione S-transferase omega 1 |
|  | GSTP1 | glutathione S-transferase pi 1 |
|  | GSTT1 | glutathione S-transferase theta 1 |
|  | GSTT2 | glutathione S-transferase theta 2 (gene/pseudogene) |
|  | GTF3C4 | general transcription factor IIIC, polypeptide 4, 90kDa |
|  | GULP1 | GULP, engulfment adaptor PTB domain containing 1 |
|  | GYS2 | glycogen synthase 2 (liver) |
|  | HAL | histidine ammonia-lyase |
|  | HCRTR1 | hypocretin (orexin) receptor 1 |
|  | HDAC10 | histone deacetylase 10 |
|  | HDAC3 | histone deacetylase 3 |
|  | HDAC4 | histone deacetylase 4 |
|  | HINT1 | histidine triad nucleotide binding protein 1 |
|  | HIPK3 | homeodomain interacting protein kinase 3 |
|  | HIST1H2AG | histone cluster 1, H2ag |
|  | HIST1H2BJ | histone cluster 1, H2bj |
|  | HLA-A | major histocompatibility complex, class I, A |
|  | HLA-B | major histocompatibility complex, class I, B |
|  | HLA-DQA1 | major histocompatibility complex, class II, DQ alpha 1 |
|  | HLA-DQB1 | major histocompatibility complex, class II, DQ beta 1 |
|  | HLA-DRB1 | major histocompatibility complex, class II, DR beta 1 |
|  | HLA-E | major histocompatibility complex, class I, E |
|  | HMBS | hydroxymethylbilane synthase |
|  | HOMER2 | homer scaffolding protein 2 |
|  | HP | haptoglobin |
|  | HPGDS | hematopoietic prostaglandin D synthase |
|  | HRH1 | histamine receptor H1 |
|  | HRH2 | histamine receptor H2 |
|  | HRH3 | histamine receptor H3 |
|  | HS3ST2 | heparan sulfate (glucosamine) 3-O-sulfotransferase 2 |
|  | HS3ST3A1 | heparan sulfate (glucosamine) 3-O-sulfotransferase 3A1 |
|  | HSPA1A | heat shock 70kDa protein 1A |
|  | HSPA1B | heat shock 70kDa protein 1B |
|  | HSPA1L | heat shock 70kDa protein 1-like |
|  | HTR1A | 5-hydroxytryptamine (serotonin) receptor 1A, G protein-coupled |
|  | HTR1B | 5-hydroxytryptamine (serotonin) receptor 1B, G protein-coupled |
|  | HTR1D | 5-hydroxytryptamine (serotonin) receptor 1D, G protein-coupled |
|  | HTR2A | 5-hydroxytryptamine (serotonin) receptor 2A, G protein-coupled |
|  | HTR2C | 5-hydroxytryptamine (serotonin) receptor 2C, G protein-coupled |
|  | HTR3A | 5-hydroxytryptamine (serotonin) receptor 3A, ionotropic |
|  | HTR3B | 5-hydroxytryptamine (serotonin) receptor 3B, ionotropic |
|  | HTR3C | 5-hydroxytryptamine (serotonin) receptor 3C, ionotropic |
|  | HTR3D | 5-hydroxytryptamine (serotonin) receptor 3D, ionotropic |
|  | HTR3E | 5-hydroxytryptamine (serotonin) receptor 3E, ionotropic |
|  | HTR4 | 5-hydroxytryptamine (serotonin) receptor 4, G protein-coupled |
|  | HTR5A | 5-hydroxytryptamine (serotonin) receptor 5A, G protein-coupled |
|  | HTR6 | 5-hydroxytryptamine (serotonin) receptor 6, G protein-coupled |
|  | HTR7 | 5-hydroxytryptamine (serotonin) receptor 7, adenylate cyclase-coupled |
|  | HTT | huntingtin |
|  | ICAM1 | intercellular adhesion molecule 1 |
|  | IL10 | interleukin 10 |
|  | IL12B | interleukin 12B |
|  | IL1A | interleukin 1, alpha |
|  | IL1B | interleukin 1, beta |
|  | IL1R1 | interleukin 1 receptor, type I |
|  | IL1RN | interleukin 1 receptor antagonist |
|  | IL2 | interleukin 2 |
|  | IL2RB | interleukin 2 receptor, beta |
|  | IL3 | interleukin 3 |
|  | IL3RA | interleukin 3 receptor, alpha (low affinity) |
|  | IL4 | interleukin 4 |
|  | IL5 | interleukin 5 |
|  | IL6 | interleukin 6 |
|  | IL6R | interleukin 6 receptor |
|  | IL9 | interleukin 9 |
|  | IMPA2 | inositol(myo)-1(or 4)-monophosphatase 2 |
|  | IMPDH2 | IMP (inosine 5'-monophosphate) dehydrogenase 2 |
|  | INSIG2 | insulin induced gene 2 |
|  | ITGA3 | integrin, alpha 3 (antigen CD49C, alpha 3 subunit of VLA-3 receptor) |
|  | ITIH1 | inter-alpha-trypsin inhibitor heavy chain 1 |
|  | ITIH4 | inter-alpha-trypsin inhibitor heavy chain family, member 4 |
|  | JAG2 | jagged 2 |
|  | JARID2 | jumonji, AT rich interactive domain 2 |
|  | KAT5 | K(lysine) acetyltransferase 5 |
|  | KCNH1 | potassium channel, voltage gated eag related subfamily H, member 1 |
|  | KCNH2 | potassium channel, voltage gated eag related subfamily H, member 2 |
|  | KCNH5 | potassium channel, voltage gated eag related subfamily H, member 5 |
|  | KCNJ10 | potassium channel, inwardly rectifying subfamily J, member 10 |
|  | KCNN3 | potassium channel, calcium activated intermediate/small conductance subfamily N alpha, member 3 |
|  | KDM4C | lysine (K)-specific demethylase 4C |
|  | KIAA0391 | KIAA0391 |
|  | KLF5 | Kruppel-like factor 5 (intestinal) |
|  | KMT2C | lysine (K)-specific methyltransferase 2C |
|  | KYNU | kynureninase |
|  | L1CAM | L1 cell adhesion molecule |
|  | LAMB2 | laminin, beta 2 (laminin S) |
|  | LEP | leptin |
|  | LIF | leukemia inhibitory factor |
|  | LIPF | lipase, gastric |
|  | LMX1A | LIM homeobox transcription factor 1, alpha |
|  | LMX1B | LIM homeobox transcription factor 1, beta |
|  | LPL | lipoprotein lipase |
|  | LRP8 | low density lipoprotein receptor-related protein 8, apolipoprotein e receptor |
|  | LSM1 | LSM1, U6 small nuclear RNA associated |
|  | LTA | lymphotoxin alpha |
|  | MACROD2 | MACRO domain containing 2 |
|  | MAD1L1 | MAD1 mitotic arrest deficient-like 1 (yeast) |
|  | MAG | myelin associated glycoprotein |
|  | MAOA | monoamine oxidase A |
|  | MAOB | monoamine oxidase B |
|  | MAP1A | microtubule-associated protein 1A |
|  | MAP1B | microtubule-associated protein 1B |
|  | MAP4 | microtubule-associated protein 4 |
|  | MAPK14 | mitogen-activated protein kinase 14 |
|  | MAPKAPK3 | mitogen-activated protein kinase-activated protein kinase 3 |
|  | MAPT | microtubule-associated protein tau |
|  | MBP | myelin basic protein |
|  | MC2R | melanocortin 2 receptor (adrenocorticotropic hormone) |
|  | MCTP2 | multiple C2 domains, transmembrane 2 |
|  | MDGA1 | MAM domain containing glycosylphosphatidylinositol anchor 1 |
|  | MED12 | mediator complex subunit 12 |
|  | MED15 | mediator complex subunit 15 |
|  | MEGF10 | multiple EGF-like-domains 10 |
|  | MET | MET proto-oncogene, receptor tyrosine kinase |
|  | MKLN1 | muskelin 1, intracellular mediator containing kelch motifs |
|  | MLEC | malectin |
|  | MMP9 | matrix metallopeptidase 9 |
|  | MPC2 | mitochondrial pyruvate carrier 2 |
|  | MSRA | methionine sulfoxide reductase A |
|  | MST1R | macrophage stimulating 1 receptor |
|  | MT-ATP6 | ATP synthase F0 subunit 6 |
|  | MTHFR | methylenetetrahydrofolate reductase (NAD(P)H) |
|  | MYH9 | myosin, heavy chain 9, non-muscle |
|  | MYLIP | myosin regulatory light chain interacting protein |
|  | MYO9B | myosin IXB |
|  | MYT1L | myelin transcription factor 1-like |
|  | NALCN | sodium leak channel, non selective |
|  | NBEAL2 | neurobeachin-like 2 |
|  | NDE1 | nudE neurodevelopment protein 1 |
|  | NDEL1 | nudE neurodevelopment protein 1-like 1 |
|  | NEFH | neurofilament, heavy polypeptide |
|  | NELFCD | negative elongation factor complex member C/D |
|  | NEUROG1 | neurogenin 1 |
|  | NFKB1 | nuclear factor of kappa light polypeptide gene enhancer in B-cells 1 |
|  | NFKBIL1 | nuclear factor of kappa light polypeptide gene enhancer in B-cells inhibitor-like 1 |
|  | NINJ2 | ninjurin 2 |
|  | NKAPL | NFKB activating protein-like |
|  | NOS1 | nitric oxide synthase 1 (neuronal) |
|  | NOS1AP | nitric oxide synthase 1 (neuronal) adaptor protein |
|  | NOS3 | nitric oxide synthase 3 (endothelial cell) |
|  | NOTCH1 | notch 1 |
|  | NOTCH2 | notch 2 |
|  | NOTCH3 | notch 3 |
|  | NOTCH4 | notch 4 |
|  | NOVA1 | neuro-oncological ventral antigen 1 |
|  | NPAS3 | neuronal PAS domain protein 3 |
|  | NPTN | neuroplastin |
|  | NPY | neuropeptide Y |
|  | NQO1 | NAD(P)H dehydrogenase, quinone 1 |
|  | NQO2 | NAD(P)H dehydrogenase, quinone 2 |
|  | NR3C1 | nuclear receptor subfamily 3, group C, member 1 (glucocorticoid receptor) |
|  | NR3C2 | nuclear receptor subfamily 3, group C, member 2 |
|  | NR4A2 | nuclear receptor subfamily 4, group A, member 2 |
|  | NRCAM | neuronal cell adhesion molecule |
|  | NREP | neuronal regeneration related protein |
|  | NRG1 | neuregulin 1 |
|  | NRG3 | neuregulin 3 |
|  | NRGN | neurogranin (protein kinase C substrate, RC3) |
|  | NRN1 | neuritin 1 |
|  | NRP1 | neuropilin 1 |
|  | NRXN1 | neurexin 1 |
|  | NRXN3 | neurexin 3 |
|  | NSF | N-ethylmaleimide-sensitive factor |
|  | NT5C2 | 5'-nucleotidase, cytosolic II |
|  | NTAN1 | N-terminal asparagine amidase |
|  | NTF3 | neurotrophin 3 |
|  | NTNG1 | netrin G1 |
|  | NTRK2 | neurotrophic tyrosine kinase, receptor, type 2 |
|  | NTRK3 | neurotrophic tyrosine kinase, receptor, type 3 |
|  | NTS | neurotensin |
|  | NTSR1 | neurotensin receptor 1 (high affinity) |
|  | NUDT6 | nudix (nucleoside diphosphate linked moiety X)-type motif 6 |
|  | NUDT9P1 | nudix (nucleoside diphosphate linked moiety X)-type motif 9 pseudogene 1 |
|  | OFCC1 | orofacial cleft 1 candidate 1 |
|  | OLR1 | oxidized low density lipoprotein (lectin-like) receptor 1 |
|  | OPCML | opioid binding protein/cell adhesion molecule-like |
|  | OPRM1 | opioid receptor, mu 1 |
|  | P2RX7 | purinergic receptor P2X, ligand gated ion channel, 7 |
|  | PADI2 | peptidyl arginine deiminase, type II |
|  | PADI4 | peptidyl arginine deiminase, type IV |
|  | PAFAH1B1 | platelet-activating factor acetylhydrolase 1b, regulatory subunit 1 (45kDa) |
|  | PAH | phenylalanine hydroxylase |
|  | PAICS | phosphoribosylaminoimidazole carboxylase, phosphoribosylaminoimidazole succinocarboxamide synthetase |
|  | PAK2 | p21 protein (Cdc42/Rac)-activated kinase 2 |
|  | PAK3 | p21 protein (Cdc42/Rac)-activated kinase 3 |
|  | PAWR | PRKC, apoptosis, WT1, regulator |
|  | PAX6 | paired box 6 |
|  | PCDH12 | protocadherin 12 |
|  | PCDHA3 | protocadherin alpha 3 |
|  | PCDHA@ | protocadherin alpha cluster, complex locus |
|  | PCDHB11 | protocadherin beta 11 |
|  | PCK1 | phosphoenolpyruvate carboxykinase 1 (soluble) |
|  | PCM1 | pericentriolar material 1 |
|  | PCNT | pericentrin |
|  | PDE4B | phosphodiesterase 4B, cAMP-specific |
|  | PDE4D | phosphodiesterase 4D, cAMP-specific |
|  | PDE7B | phosphodiesterase 7B |
|  | PDLIM5 | PDZ and LIM domain 5 |
|  | PDYN | prodynorphin |
|  | PEA15 | phosphoprotein enriched in astrocytes 15 |
|  | PEMT | phosphatidylethanolamine N-methyltransferase |
|  | PER3 | period circadian clock 3 |
|  | PGBD1 | piggyBac transposable element derived 1 |
|  | PGPEP1 | pyroglutamyl-peptidase I |
|  | PHGDH | phosphoglycerate dehydrogenase |
|  | PI4KA | phosphatidylinositol 4-kinase, catalytic, alpha |
|  | PICK1 | protein interacting with PRKCA 1 |
|  | PIK3C2A | phosphatidylinositol-4-phosphate 3-kinase, catalytic subunit type 2 alpha |
|  | PIK3C2G | phosphatidylinositol-4-phosphate 3-kinase, catalytic subunit type 2 gamma |
|  | PIK3C3 | phosphatidylinositol 3-kinase, catalytic subunit type 3 |
|  | PIK3CA | phosphatidylinositol-4,5-bisphosphate 3-kinase, catalytic subunit alpha |
|  | PIK3CB | phosphatidylinositol-4,5-bisphosphate 3-kinase, catalytic subunit beta |
|  | PIP4K2A | phosphatidylinositol-5-phosphate 4-kinase, type II, alpha |
|  | PIP5K1B | phosphatidylinositol-4-phosphate 5-kinase, type I, beta |
|  | PITX3 | paired-like homeodomain 3 |
|  | PLA2G1B | phospholipase A2, group IB (pancreas) |
|  | PLA2G2A | phospholipase A2, group IIA (platelets, synovial fluid) |
|  | PLA2G4A | phospholipase A2, group IVA (cytosolic, calcium-dependent) |
|  | PLA2G4B | phospholipase A2, group IVB (cytosolic) |
|  | PLA2G4C | phospholipase A2, group IVC (cytosolic, calcium-independent) |
|  | PLA2G4D | phospholipase A2, group IVD (cytosolic) |
|  | PLA2G6 | phospholipase A2, group VI (cytosolic, calcium-independent) |
|  | PLA2G7 | phospholipase A2, group VII (platelet-activating factor acetylhydrolase, plasma) |
|  | PLAA | phospholipase A2-activating protein |
|  | PLCB2 | phospholipase C, beta 2 |
|  | PLXNA2 | plexin A2 |
|  | PLXNB1 | plexin B1 |
|  | PNPLA8 | patatin-like phospholipase domain containing 8 |
|  | PNPO | pyridoxamine 5'-phosphate oxidase |
|  | POM121L2 | POM121 transmembrane nucleoporin-like 2 |
|  | PON1 | paraoxonase 1 |
|  | POU3F2 | POU class 3 homeobox 2 |
|  | PPARD | peroxisome proliferator-activated receptor delta |
|  | PPARG | peroxisome proliferator-activated receptor gamma |
|  | PPP1R1B | protein phosphatase 1, regulatory (inhibitor) subunit 1B |
|  | PPP1R36 | protein phosphatase 1, regulatory subunit 36 |
|  | PPP2R2B | protein phosphatase 2, regulatory subunit B, beta |
|  | PPP3CC | protein phosphatase 3, catalytic subunit, gamma isozyme |
|  | PPT1 | palmitoyl-protein thioesterase 1 |
|  | PRKAR2A | protein kinase, cAMP-dependent, regulatory, type II, alpha |
|  | PRKCA | protein kinase C, alpha |
|  | PRMT7 | protein arginine methyltransferase 7 |
|  | PRNP | prion protein |
|  | PRODH | proline dehydrogenase (oxidase) 1 |
|  | PRSS16 | protease, serine, 16 (thymus) |
|  | PSAP | prosaposin |
|  | PSEN1 | presenilin 1 |
|  | PSTPIP2 | proline-serine-threonine phosphatase interacting protein 2 |
|  | PTBP2 | polypyrimidine tract binding protein 2 |
|  | PTGFRN | prostaglandin F2 receptor inhibitor |
|  | PTGS1 | prostaglandin-endoperoxide synthase 1 (prostaglandin G/H synthase and cyclooxygenase) |
|  | PTGS2 | prostaglandin-endoperoxide synthase 2 (prostaglandin G/H synthase and cyclooxygenase) |
|  | PTPRG | protein tyrosine phosphatase, receptor type, G |
|  | PTPRZ1 | protein tyrosine phosphatase, receptor-type, Z polypeptide 1 |
|  | RAPGEF6 | Rap guanine nucleotide exchange factor (GEF) 6 |
|  | RASD2 | RASD family, member 2 |
|  | RELN | reelin |
|  | RERE | arginine-glutamic acid dipeptide (RE) repeats |
|  | RET | ret proto-oncogene |
|  | RGS16 | regulator of G-protein signaling 16 |
|  | RGS2 | regulator of G-protein signaling 2 |
|  | RGS4 | regulator of G-protein signaling 4 |
|  | RGS5 | regulator of G-protein signaling 5 |
|  | RGS8 | regulator of G-protein signaling 8 |
|  | RGS9 | regulator of G-protein signaling 9 |
|  | RHD | Rh blood group, D antigen |
|  | RIT2 | Ras-like without CAAX 2 |
|  | RNLS | renalase, FAD-dependent amine oxidase |
|  | ROBO1 | roundabout, axon guidance receptor, homolog 1 (Drosophila) |
|  | ROBO2 | roundabout, axon guidance receptor, homolog 2 (Drosophila) |
|  | RPA1 | replication protein A1, 70kDa |
|  | RPGRIP1L | RPGRIP1-like |
|  | RPL5 | ribosomal protein L5 |
|  | RSRC1 | arginine/serine-rich coiled-coil 1 |
|  | RTN4 | reticulon 4 |
|  | RTN4R | reticulon 4 receptor |
|  | RUNDC3B | RUN domain containing 3B |
|  | RUNX1 | runt-related transcription factor 1 |
|  | RXRB | retinoid X receptor, beta |
|  | S100B | S100 calcium binding protein B |
|  | SAT1 | spermidine/spermine N1-acetyltransferase 1 |
|  | SDCCAG8 | serologically defined colon cancer antigen 8 |
|  | SELE | selectin E |
|  | SELENBP1 | selenium binding protein 1 |
|  | SEMA3B | sema domain, immunoglobulin domain (Ig), short basic domain, secreted, (semaphorin) 3B |
|  | SEMA3D | sema domain, immunoglobulin domain (Ig), short basic domain, secreted, (semaphorin) 3D |
|  | SEMA6C | sema domain, transmembrane domain (TM), and cytoplasmic domain, (semaphorin) 6C |
|  | SETD2 | SET domain containing 2 |
|  | SHISA5 | shisa family member 5 |
|  | SHISA9 | shisa family member 9 |
|  | SHMT1 | serine hydroxymethyltransferase 1 (soluble) |
|  | SIGMAR1 | sigma non-opioid intracellular receptor 1 |
|  | SIM1 | single-minded family bHLH transcription factor 1 |
|  | SLC12A2 | solute carrier family 12 (sodium/potassium/chloride transporter), member 2 |
|  | SLC17A1 | solute carrier family 17 (organic anion transporter), member 1 |
|  | SLC17A3 | solute carrier family 17 (organic anion transporter), member 3 |
|  | SLC17A6 | solute carrier family 17 (vesicular glutamate transporter), member 6 |
|  | SLC17A7 | solute carrier family 17 (vesicular glutamate transporter), member 7 |
|  | SLC18A1 | solute carrier family 18 (vesicular monoamine transporter), member 1 |
|  | SLC18A2 | solute carrier family 18 (vesicular monoamine transporter), member 2 |
|  | SLC1A2 | solute carrier family 1 (glial high affinity glutamate transporter), member 2 |
|  | SLC1A4 | solute carrier family 1 (glutamate/neutral amino acid transporter), member 4 |
|  | SLC1A5 | solute carrier family 1 (neutral amino acid transporter), member 5 |
|  | SLC22A4 | solute carrier family 22 (organic cation/zwitterion transporter), member 4 |
|  | SLC24A5 | solute carrier family 24 (sodium/potassium/calcium exchanger), member 5 |
|  | SLC26A6 | solute carrier family 26 (anion exchanger), member 6 |
|  | SLC35A5 | solute carrier family 35, member A5 |
|  | SLC6A2 | solute carrier family 6 (neurotransmitter transporter), member 2 |
|  | SLC6A3 | solute carrier family 6 (neurotransmitter transporter), member 3 |
|  | SLC6A4 | solute carrier family 6 (neurotransmitter transporter), member 4 |
|  | SLC6A5 | solute carrier family 6 (neurotransmitter transporter), member 5 |
|  | SLC6A9 | solute carrier family 6 (neurotransmitter transporter, glycine), member 9 |
|  | SLCO3A1 | solute carrier organic anion transporter family, member 3A1 |
|  | SLCO6A1 | solute carrier organic anion transporter family, member 6A1 |
|  | SMARCAD1 | SWI/SNF-related, matrix-associated actin-dependent regulator of chromatin, subfamily a, containing DEAD/H box 1 |
|  | SMARCC1 | SWI/SNF related, matrix associated, actin dependent regulator of chromatin, subfamily c, member 1 |
|  | SMPD1 | sphingomyelin phosphodiesterase 1, acid lysosomal |
|  | SNAP25 | synaptosomal-associated protein, 25kDa |
|  | SNAP29 | synaptosomal-associated protein, 29kDa |
|  | SOD2 | superoxide dismutase 2, mitochondrial |
|  | SPARCL1 | SPARC-like 1 (hevin) |
|  | SPRY4 | sprouty homolog 4 (Drosophila) |
|  | SPTBN1 | spectrin, beta, non-erythrocytic 1 |
|  | SRD5A1 | steroid-5-alpha-reductase, alpha polypeptide 1 (3-oxo-5 alpha-steroid delta 4-dehydrogenase alpha 1) |
|  | SREBF1 | sterol regulatory element binding transcription factor 1 |
|  | SREBF2 | sterol regulatory element binding transcription factor 2 |
|  | SRR | serine racemase |
|  | ST6GAL2 | ST6 beta-galactosamide alpha-2,6-sialyltranferase 2 |
|  | ST8SIA2 | ST8 alpha-N-acetyl-neuraminide alpha-2,8-sialyltransferase 2 |
|  | ST8SIA4 | ST8 alpha-N-acetyl-neuraminide alpha-2,8-sialyltransferase 4 |
|  | STAB1 | stabilin 1 |
|  | STT3A | STT3A, subunit of the oligosaccharyltransferase complex (catalytic) |
|  | SULT4A1 | sulfotransferase family 4A, member 1 |
|  | SYN2 | synapsin II |
|  | SYN3 | synapsin III |
|  | SYNGR1 | synaptogyrin 1 |
|  | TAAR6 | trace amine associated receptor 6 |
|  | TAC1 | tachykinin, precursor 1 |
|  | TACR3 | tachykinin receptor 3 |
|  | TAP1 | transporter 1, ATP-binding cassette, sub-family B (MDR/TAP) |
|  | TAP2 | transporter 2, ATP-binding cassette, sub-family B (MDR/TAP) |
|  | TAPBP | TAP binding protein (tapasin) |
|  | TBP | TATA box binding protein |
|  | TBX1 | T-box 1 |
|  | TC2N | tandem C2 domains, nuclear |
|  | TCF4 | transcription factor 4 |
|  | TCF7L2 | transcription factor 7-like 2 (T-cell specific, HMG-box) |
|  | TF | transferrin |
|  | TFAP2A | transcription factor AP-2 alpha (activating enhancer binding protein 2 alpha) |
|  | TGM2 | transglutaminase 2 |
|  | TH | tyrosine hydroxylase |
|  | TLR9 | toll-like receptor 9 |
|  | TNF | tumor necrosis factor |
|  | TNFRSF1A | tumor necrosis factor receptor superfamily, member 1A |
|  | TNFRSF1B | tumor necrosis factor receptor superfamily, member 1B |
|  | TNIK | TRAF2 and NCK interacting kinase |
|  | TNR | tenascin R |
|  | TNXB | tenascin XB |
|  | TP53 | tumor protein p53 |
|  | TPH1 | tryptophan hydroxylase 1 |
|  | TPH2 | tryptophan hydroxylase 2 |
|  | TRAF1 | TNF receptor-associated factor 1 |
|  | TRAF3 | TNF receptor-associated factor 3 |
|  | TRAF3IP1 | TNF receptor-associated factor 3 interacting protein 1 |
|  | TRIM26 | tripartite motif containing 26 |
|  | TSNAX | translin-associated factor X |
|  | TSPAN18 | tetraspanin 18 |
|  | TSPO | translocator protein (18kDa) |
|  | TTR | transthyretin |
|  | TUBA1A | tubulin, alpha 1a |
|  | TXNDC5 | thioredoxin domain containing 5 (endoplasmic reticulum) |
|  | UFD1L | ubiquitin fusion degradation 1 like (yeast) |
|  | UHMK1 | U2AF homology motif (UHM) kinase 1 |
|  | UNC5C | unc-5 homolog C (C. elegans) |
|  | USP4 | ubiquitin specific peptidase 4 (proto-oncogene) |
|  | UTRN | utrophin |
|  | VPS13C | vacuolar protein sorting 13 homolog C (S. cerevisiae) |
|  | VRK2 | vaccinia related kinase 2 |
|  | VSNL1 | visinin-like 1 |
|  | WIF1 | WNT inhibitory factor 1 |
|  | WNT7A | wingless-type MMTV integration site family, member 7A |
|  | WWC1 | WW and C2 domain containing 1 |
|  | WWOX | WW domain containing oxidoreductase |
|  | XBP1 | X-box binding protein 1 |
|  | XKR4 | XK, Kell blood group complex subunit-related family, member 4 |
|  | XRCC1 | X-ray repair complementing defective repair in Chinese hamster cells 1 |
|  | YWHAH | tyrosine 3-monooxygenase/tryptophan 5-monooxygenase activation protein, eta |
|  | ZBTB20 | zinc finger and BTB domain containing 20 |
|  | ZDHHC8 | zinc finger, DHHC-type containing 8 |
|  | ZNF184 | zinc finger protein 184 |
|  | ZNF74 | zinc finger protein 74 |
|  | ZNF804A | zinc finger protein 804A |
|  | SNCA | Synuclein alpha |
|  | SEPTIN5 | Septin 5 |
|  | GABRG1 | Gamma-aminobutyric acid type A receptor subunit gamma1 |
|  | NGF | Nerve growth factor |
|  | LDLRAD4 | Low density lipoprotein receptor class A domain containing 4 |
|  | SP4 | Sp4 transcription factor |
|  | DPP10 | Dipeptidyl peptidase like 10 |
|  | GABRA2 | Gamma-aminobutyric acid type A receptor subunit alpha2 |
|  | PLCB4 | Phospholipase C beta 4 |
|  | CHAT | Choline O-acetyltransferase |
|  | GABRA4 | Gamma-aminobutyric acid type A receptor subunit alpha4 |
|  | GRK3 | G protein-coupled receptor kinase 3 |
|  | GABRB1 | Gamma-aminobutyric acid type A receptor subunit beta1 |
|  | IPO5 | Importin 5 |
|  | THBS1 | Thrombospondin 1 |
|  | ACTB | Actin beta |
|  | UFD1 | Ubiquitin recognition factor in ER associated degradation 1 |
|  | CHGA | Chromogranin A |
|  | KPNA3 | Karyopherin subunit alpha 3 |
|  | SLC25A27 | Solute carrier family 25 member 27 |
|  | MCHR1 | Melanin concentrating hormone receptor 1 |
|  | PLCB1 | Phospholipase C beta 1 |
|  | TPM3 | Tropomyosin 3 |
|  | IL18 | Interleukin 18 |
|  | CAMK2B | Calcium/calmodulin dependent protein kinase II beta |
|  | H2BC11 | H2B clustered histone 11 |
|  | GNAL | G protein subunit alpha L |
|  | TFIP11 | Tuftelin interacting protein 11 |
|  | GGT1 | Gamma-glutamyltransferase 1 |
|  | YWHAB | Tyrosine 3-monooxygenase/tryptophan 5-monooxygenase activation protein beta |
|  | ATP5F1A | ATP synthase F1 subunit alpha |
|  | CFL1 | Cofilin 1 |
|  | VIPR2 | Vasoactive intestinal peptide receptor 2 |
|  | PHOX2B | Paired like homeobox 2B |
|  | GNAS | GNAS complex locus |
|  | ATP6V1B2 | ATPase H+ transporting V1 subunit B2 |
|  | MLC1 | Modulator of VRAC current 1 |
|  | EFHD2 | EF-hand domain family member D2 |
|  | PRL | Prolactin |
|  | SLC1A1 | Solute carrier family 1 member 1 |
|  | SLC1A3 | Solute carrier family 1 member 3 |
|  | APOA5 | Apolipoprotein A5 |
|  | FABP7 | Fatty acid binding protein 7 |
|  | CKB | Creatine kinase B |
|  | CNTNAP5 | Contactin associated protein family member 5 |
|  | SIRPB1 | Signal regulatory protein beta 1 |
|  | INSIG1 | Insulin induced gene 1 |
|  | NEFM | Neurofilament medium chain |
|  | APOA1 | Apolipoprotein A1 |
|  | PGAM1 | Phosphoglycerate mutase 1 |
|  | LIFR | LIF receptor subunit alpha |
|  | SYN1 | Synapsin I |
|  | ANK2 | Ankyrin 2 |
|  | DNAJB1 | DnaJ heat shock protein family (Hsp40) member B1 |
|  | SNX8 | Sorting nexin 8 |
|  | GSTA1 | Glutathione S-transferase alpha 1 |
|  | PPIA | Peptidylprolyl isomerase A |
|  | TRAK1 | Trafficking kinesin protein 1 |
|  | CSNK2A1 | Casein kinase 2 alpha 1 |
|  | ADAM12 | ADAM metallopeptidase domain 12 |
|  | BRMS1 | BRMS1 transcriptional repressor and anoikis regulator |
|  | SYNJ1 | Synaptojanin 1 |
|  | VDR | Vitamin D receptor |
|  | DGCR6 | DiGeorge syndrome critical region gene 6 |
|  | HK1 | Hexokinase 1 |
|  | IMMT | Inner membrane mitochondrial protein |
|  | CALM1 | Calmodulin 1 |
|  | FTL | Ferritin light chain |
|  | TUBB2A | Tubulin beta 2A class IIa |
|  | ERBB2 | Erb-b2 receptor tyrosine kinase 2 |
|  | FMR1 | FMRP translational regulator 1 |
|  | QKI | QKI, KH domain containing RNA binding |
|  | B2M | Beta-2-microglobulin |
|  | GPRASP2 | G protein-coupled receptor associated sorting protein 2 |
|  | LEPR | Leptin receptor |
|  | NTNG2 | Netrin G2 |
|  | DKK4 | Dickkopf WNT signaling pathway inhibitor 4 |
|  | NCAM1 | Neural cell adhesion molecule 1 |
|  | YWHAE | Tyrosine 3-monooxygenase/tryptophan 5-monooxygenase activation protein epsilon |
|  | HSPA8 | Heat shock protein family A (Hsp70) member 8 |
|  | MAP6 | Microtubule associated protein 6 |
|  | CREBBP | CREB binding protein |
|  | OXTR | Oxytocin receptor |
|  | GRIK2 | Glutamate ionotropic receptor kainate type subunit 2 |
|  | GRM1 | Glutamate metabotropic receptor 1 |
|  | RNH1 | Ribonuclease/angiogenin inhibitor 1 |
|  | PCMT1 | Protein-L-isoaspartate (D-aspartate) O-methyltransferase |
|  | DAOA-AS1 | DAOA antisense RNA 1 |
|  | NEFL | Neurofilament light chain |
|  | TPI1 | Triosephosphate isomerase 1 |
|  | YWHAG | Tyrosine 3-monooxygenase/tryptophan 5-monooxygenase activation protein gamma |
|  | GRIN3B | GRIN3B – glutamate ionotropic receptor NMDA type subunit 3B |
|  | DPYD | Dihydropyrimidine dehydrogenase |
|  | GABRR1 | Gamma-aminobutyric acid type A receptor subunit rho1 |
|  | GABRR2 | Gamma-aminobutyric acid type A receptor subunit rho2 |
|  | SYP | Synaptophysin |
|  | OGG1 | 8-oxoguanine DNA glycosylase |
|  | UCHL1 | Ubiquitin C-terminal hydrolase L1 |
|  | CNTNAP2 | Contactin associated protein 2 |
|  | ACHE | Acetylcholinesterase (Cartwright blood group) |
|  | GFAP | Glial fibrillary acidic protein |
|  | ADM | Adrenomedullin |
|  | VLDLR | Very low density lipoprotein receptor |
|  | MMP16 | Matrix metallopeptidase 16 |
|  | MAP2 | Microtubule associated protein 2 |
|  | STXBP1 | Syntaxin binding protein 1 |
|  | GRN | Granulin precursor |
|  | MAGI2 | Membrane associated guanylate kinase, WW and PDZ domain containing 2 |
|  | PLP1 | Proteolipid protein 1 |
|  | MTOR | Mechanistic target of rapamycin kinase |
|  | CHRNA6 | Cholinergic receptor nicotinic alpha 6 subunit |
|  | FYN | FYN proto-oncogene, Src family tyrosine kinase |
|  | GLO1 | Glyoxalase I |
|  | DGCR8 | DGCR8 microprocessor complex subunit |
|  | BACE1 | Beta-secretase 1 |
|  | PRKCG | Protein kinase C gamma |
|  | AADAT | Aminoadipate aminotransferase |
|  | TUBB | Tubulin beta class I |
|  | IGF1 | Insulin like growth factor 1 |
|  | NR4A1 | Nuclear receptor subfamily 4 group A member 1 |
|  | ARC | Activity regulated cytoskeleton associated protein |
|  | OLIG2 | Oligodendrocyte transcription factor 2 |
|  | SP1 | Sp1 transcription factor |
|  | MC4R | Melanocortin 4 receptor |
|  | MECP2 | Methyl-CpG binding protein 2 |
|  | ASCL1 | Achaete-scute family bHLH transcription factor 1 |
|  | NLGN1 | Neuroligin 1 |
|  | NPSR1 | Neuropeptide S receptor 1 |
|  | DMD | Dystrophin |
|  | FABP3 | Fatty acid binding protein 3 |
|  | GABRB3 | Gamma-aminobutyric acid type A receptor subunit beta3 |
|  | DLG2 | Discs large MAGUK scaffold protein 2 |
|  | PGK1 | Phosphoglycerate kinase 1 |
|  | SLC1A6 | Solute carrier family 1 member 6 |
|  | NTRK1 | Neurotrophic receptor tyrosine kinase 1 |
|  | NCAN | Neurocan |
|  | OXT | Oxytocin/neurophysin I prepropeptide |
|  | CRP | C-reactive protein |
|  | FAS | Fas cell surface death receptor |
|  | BLOC1S1 | Biogenesis of lysosomal organelles complex 1 subunit 1 |
|  | GRIP1 | Glutamate receptor interacting protein 1 |
|  | GNB1 | G protein subunit beta 1 |
|  | CRYM | Crystallin mu |
|  | PVALB | Parvalbumin |
|  | MPO | Myeloperoxidase |
|  | DBP | D-box binding PAR bZIP transcription factor |
|  | SMARCA2 | SWI/SNF related, matrix associated, actin dependent regulator of chromatin, subfamily a, member 2 |
|  | MICB | MHC class I polypeptide-related sequence B |
|  | CHRM4 | Cholinergic receptor muscarinic 4 |
|  | ANKS1B | Ankyrin repeat and sterile alpha motif domain containing 1B |
|  | PBRM1 | Polybromo 1 |
|  | HOMER1 | Homer scaffold protein 1 |
|  | GRIA1 | Glutamate ionotropic receptor AMPA type subunit 1 |
|  | GABRG3 | Gamma-aminobutyric acid type A receptor subunit gamma3 |
|  | PPP3CA | Protein phosphatase 3 catalytic subunit alpha |
|  | NLGN4X | Neuroligin 4 X-linked |
|  | SOX10 | SRY-box transcription factor 10 |
|  | ARNTL | Aryl hydrocarbon receptor nuclear translocator like |
|  | MACF1 | Microtubule actin crosslinking factor 1 |
|  | UCP2 | Uncoupling protein 2 |
|  | PRKAA2 | Protein kinase AMP-activated catalytic subunit alpha 2 |
|  | CNTN4 | Contactin 4 |
|  | GAPDH | Glyceraldehyde-3-phosphate dehydrogenase |
|  | IL4R | Interleukin 4 receptor |
|  | PINK1 | PTEN induced kinase 1 |
|  | VIM | Vimentin |
|  | GABRA3 | Gamma-aminobutyric acid type A receptor subunit alpha3 |
|  | QDPR | Quinoid dihydropteridine reductase |
|  | CPO | Carboxypeptidase O |
|  | ALDH1L1 | Aldehyde dehydrogenase 1 family member L1 |
|  | SMARCE1 | SWI/SNF related, matrix associated, actin dependent regulator of chromatin, subfamily e, member 1 |
|  | CMYA5 | Cardiomyopathy associated 5 |
|  | TSPAN7 | Tetraspanin 7 |
|  | APP | Amyloid beta precursor protein |
|  | GRIN2C | Glutamate ionotropic receptor NMDA type subunit 2C |
|  | TKT | Transketolase |
|  | TACR1 | Tachykinin receptor 1 |
|  | KMO | Kynurenine 3-monooxygenase |
|  | UBE2N | Ubiquitin conjugating enzyme E2 N |
|  | CREB1 | cAMP responsive element binding protein 1 |
|  | TUBA1B | Tubulin alpha 1b |
|  | DNMT1 | DNA methyltransferase 1 |
|  | EIF3H | Eukaryotic translation initiation factor 3 subunit H |
|  | VAMP2 | Vesicle associated membrane protein 2 |
|  | CTNNB1 | Catenin beta 1 |
|  | PPP3R1 | Protein phosphatase 3 regulatory subunit B, alpha |
|  | APH1B | Aph-1 homolog B, gamma-secretase subunit |
|  | MOG | Myelin oligodendrocyte glycoprotein |
|  | SLC9A3R1 | SLC9A3 regulator 1 |
|  | PKNOX2 | PBX/knotted 1 homeobox 2 |
|  | ZNF365 | Zinc finger protein 365 |
|  | CALR | Calreticulin |
|  | YWHAZ | Tyrosine 3-monooxygenase/tryptophan 5-monooxygenase activation protein zeta |
|  | PTGDS | Prostaglandin D2 synthase |
|  | KALRN | Kalirin RhoGEF kinase |
|  | PPP1R9B | Protein phosphatase 1 regulatory subunit 9B |
|  | ITIH3 | Inter-alpha-trypsin inhibitor heavy chain 3 |
|  | DDR1 | Discoidin domain receptor tyrosine kinase 1 |
|  | TIMELESS | Timeless circadian regulator |
|  | UBC | Biquitin C |
|  | CAMKK2 | Calcium/calmodulin dependent protein kinase kinase 2 |
|  | ACTG1 | Actin gamma 1 |
|  | ACTN2 | Actinin alpha 2 |
|  | SYT11 | Synaptotagmin 11 |
|  | PER2 | Period circadian regulator 2 |
|  | DBNDD1 | Dysbindin domain containing 1 |
|  | POMC | Proopiomelanocortin |
|  | CPLX1 | CPLX1 – complexin 1 |
|  | DNMT3A | DNA methyltransferase 3 alpha |
|  | SOD1 | Superoxide dismutase 1 |
|  | SPTAN1 | Spectrin alpha, non-erythrocytic 1 |
|  | NCS1 | Neuronal calcium sensor 1 |
|  | CNTN5 | CNTN5 – contactin 5 |
|  | CTSK | Cathepsin K |
|  | CEP63 | Centrosomal protein 63 |
|  | UGT1A4 | UDP glucuronosyltransferase family 1 member A4 |
|  | UQCRC1 | Ubiquinol-cytochrome c reductase core protein 1 |
|  | SLC32A1 | Solute carrier family 32 member 1 |
|  | ACO2 | Aconitase 2 |
|  | PEBP1 | Phosphatidylethanolamine binding protein 1 |
|  | PNOC | Prepronociceptin |
|  | ERMN | Ermin |
|  | PDGFRB | Platelet derived growth factor receptor beta |
|  | EPB41L1 | Erythrocyte membrane protein band 4.1 like 1 |
|  | ACACB | Acetyl-CoA carboxylase beta |
|  | IL1RAPL1 | Interleukin 1 receptor accessory protein like 1 |
|  | PPFIA2 | PTPRF interacting protein alpha 2 |
|  | CAMK2A | Calcium/calmodulin dependent protein kinase II alpha |
|  | ATP6V1A | ATPase H+ transporting V1 subunit A |
|  | NDUFV2 | NADH:ubiquinone oxidoreductase core subunit V2 |
|  | FABP5 | Fatty acid binding protein 5 |
|  | WNT1 | Wnt family member 1 |
|  | SEMA3A | Semaphorin 3A |
|  | SEPTIN4 | Septin 4 |
|  | SYNE1 | Spectrin repeat containing nuclear envelope protein 1 |
|  | BRCA2 | BRCA2 DNA repair associated |
|  | APOD | Apolipoprotein D |
|  | MTNR1A | Melatonin receptor 1A |
|  | IRS4 | Insulin receptor substrate 4 |
|  | HCAR2 | Hydroxycarboxylic acid receptor 2 |
|  | GRIK1 | Glutamate ionotropic receptor kainate type subunit 1 |
|  | RBFOX1 | RNA binding fox-1 homolog 1 |
|  | ACACA | Acetyl-CoA carboxylase alpha |
|  | ALDOC | Aldolase, fructose-bisphosphate C |
|  | GAP43 | Growth associated protein 43 |
|  | BCHE | Butyrylcholinesterase |
|  | HSPA5 | Heat shock protein family A (Hsp70) member 5 |
|  | LRRTM1 | Leucine rich repeat transmembrane neuronal 1 |
|  | STX1A | Syntaxin 1A |
|  | MAGI1 | Membrane associated guanylate kinase, WW and PDZ domain containing 1 |
|  | SHANK3 | SH3 and multiple ankyrin repeat domains 3 |

| Supplementary.Table.2 All genes associated with Obsessive-compulsive disorder extracted from GeneWeaver and Harmonizome. | | |
| --- | --- | --- |
| Index | **Genes symbol** | **Gene full name** |
|  | SLC6A4 | Solute carrier family 6 member 4 |
|  | GRIK2 | Glutamate ionotropic receptor kainate type subunit 2 |
|  | MAOA | Monoamine oxidase A |
|  | DRD4 | Dopamine receptor D4 |
|  | COMT | Catechol-O-methyltransferase |
|  | EAAT3 | Solute carrier family 1 member 1 |
|  | GRIN2B | Glutamate ionotropic receptor NMDA type subunit 2B |
|  | HTR2A | 5-hydroxytryptamine receptor 2A |
|  | BDNF | Brain derived neurotrophic factor |
|  | HTR1B | 5-hydroxytryptamine receptor 1B |
|  | TPH2 | Tryptophan hydroxylase 2 |
|  | OLIG2 | Oligodendrocyte transcription factor 2 |
|  | GABBR1 | Gamma-aminobutyric acid type B receptor subunit 1 |
|  | NTRK2 | Neurotrophic receptor tyrosine kinase 2 |
|  | MOG | Myelin oligodendrocyte glycoprotein |
|  | SAPAP3 | DLG associated protein 3 |
|  | DRD3 | Dopamine receptor D3 |
|  | TNFA | Tumor necrosis factor |
|  | ESR1 | Estrogen receptor 1 |
|  | HTR2B | 5-hydroxytryptamine receptor 2B |
|  | NTRK3 | Neurotrophic receptor tyrosine kinase 3 |
|  | HTR3A | 5-hydroxytryptamine receptor 3A |
|  | FOXD4 | Forkhead box D4 |
|  | DLGAP1 | DLG associated protein 1 |
|  | RYR3 | Ryanodine receptor 3 |
|  | PBX1 | PBX homeobox 1 |
|  | LMX1A | LIM homeobox transcription factor 1 alpha |
|  | MEIS2 | Meis homeobox 2 |
|  | PTPRD | Protein tyrosine phosphatase receptor type D |
|  | mTOR | Mechanistic target of rapamycin kinase |
|  | DRD2 | Dopamine receptor D2 |
|  | HCN4 | Hyperpolarization activated cyclic nucleotide gated potassium channel 4 |
|  | FKBP5 | FKBP prolyl isomerase 5 |
|  | HTR1D | 5-hydroxytryptamine receptor 1D |
|  | SLITRK3 | SLIT and NTRK like family member 3 |
|  | SLITRK5 | SLIT and NTRK like family member 5 |
|  | SLITRK1 | SLIT and NTRK like family member 1 |
|  | SLC18A1 | Solute carrier family 18 member A1 |
|  | GAD1 | Glutamate decarboxylase 1 |
|  | GAD2 | Glutamate decarboxylase 2 |
|  | COL27A1 | Collagen type XXVII alpha 1 chain |
|  | SLC22A3 | Solute carrier family 22 member 3 |
|  | 5HT2C | 5-hydroxytryptamine receptor 2C |
|  | SLC6A3 | Solute carrier family 6 member 3 |
|  | GRIK3 | Glutamate ionotropic receptor kainate type subunit 3 |
|  | CDH9 | Cadherin 9 |
|  | NEUROD6 | Neuronal differentiation 6 |
|  | SV2A | Synaptic vesicle glycoprotein 2A |
|  | GRIA4 | Glutamate ionotropic receptor AMPA type subunit 4 |
|  | SLC1A2 | Solute carrier family 1 member 2 |
|  | IQCK | IQ motif containing K |
|  | C16orf88 | Lysine rich nucleolar protein 1) |
|  | OFCC1 | Orofacial cleft 1 candidate 1 |
|  | CDH10 | Cadherin 10 |
|  | ESR2 | Estrogen receptor 2 |
|  | BTBD3 | BTB domain containing 3 |
|  | FAIM2 | Fas apoptotic inhibitory molecule 2 |
|  | ADCY8 | Adenylate cyclase 8 |
|  | OPRM1 | Opioid receptor mu 1 |
|  | ISM1 | Isthmin 1 |
|  | NRXN1 | Neurexin 1 |
|  | CTTNBP2 | Cortactin binding protein 2 |
|  | REEP3 | Receptor accessory protein 3 |
|  | CCKBR | Cholecystokinin B receptor |
|  | CHRNA10 | Cholinergic receptor nicotinic alpha 10 subunit |
|  | KCNH5 | Potassium voltage-gated channel subfamily H member 5 |
|  | KCNK10 | Potassium two pore domain channel subfamily K member 10 |
|  | NRXN3 | Neurexin 3 |
|  | OXTR | Oxytocin receptor |
|  | TPH1 | Tryptophan hydroxylase 1 |
|  | NTRK1 | Neurotrophic receptor tyrosine kinase 1 |
|  | NFKBIL1 | NFKB inhibitor like 1 |
|  | AQP2 | Aquaporin 2 |
|  | HACE1 | HECT domain and ankyrin repeat containing E3 ubiquitin protein ligase 1 |
|  | MZT1 | Mitotic spindle organizing protein 1 |
|  | DACH1 | Dachshund family transcription factor 1 |
|  | FUT2 | Fucosyltransferase 2 |
|  | SGCE | Sarcoglycan epsilon |
|  | CYP2D6 | Cytochrome P450 family 2 subfamily D member 6 |
|  | CDH2 | Cadherin 2 |
|  | PCDH10 | Protocadherin 10 |
|  | GPC6 | Glypican 6 |
|  | GABRG2 | Gamma-aminobutyric acid type A receptor subunit gamma2 |
|  | NOS1AP | Nitric oxide synthase 1 adaptor protein |
|  | nNOS | NOS1= Nitric oxide synthase 1 |
|  | CNTNAP2 | Contactin associated protein 2 |
|  | NPSR1 | Neuropeptide S receptor 1 |
|  | GRIA2 | Glutamate ionotropic receptor AMPA type subunit 2 |
|  | DNM3 | Dynamin 3 |
|  | PKM | Pyruvate kinase M1/2 |
|  | Grm5 | Glutamate metabotropic receptor 5 |
|  | BDKRB2 | Bradykinin receptor B2 |
|  | CYP2E1 | Cytochrome P450 family 2 subfamily E member 1 |
|  | CNR1 | Cannabinoid receptor 1 |
|  | DLG4 | Discs large MAGUK scaffold protein 4 |
|  | HTR1A | 5-hydroxytryptamine receptor 1A |
|  | FOS | Fos proto-oncogene, AP-1 transcription factor subunit |
|  | MNDA | Myeloid cell nuclear differentiation antigen |
|  | RGS4 | Regulator of G protein signaling 4 |
|  | RORB | RAR related orphan receptor B |
|  | CHCHD2 | Coiled-coil-helix-coiled-coil-helix domain containing 2 |
|  | MAOB | Monoamine oxidase B |
|  | CYP2C19 | Cytochrome P450 family 2 subfamily C member 19 |
|  | DRD1 | Dopamine receptor D1 |
|  | ANKK1 | Ankyrin repeat and kinase domain containing 1 |
|  | BCYRN1 | Brain cytoplasmic RNA 1 |
|  | BCOR | BCL6 corepressor |
|  | FGF13 | Fibroblast growth factor 13 |
|  | HLA-DRB1 | Major histocompatibility complex, class II, DR beta 1 |
|  | ARX | Aristaless related homeobox |
|  | ARHGAP6 | Rho GTPase activating protein 6 |
|  | G6PD | Glucose-6-phosphate dehydrogenase |
|  | PQBP1 | Polyglutamine binding protein 1 |
|  | TIMM17B | Translocase of inner mitochondrial membrane 17B |
|  | IKBKG | Inhibitor of nuclear factor kappa B kinase regulatory subunit gamma |
|  | EFNB1 | Ephrin B1 |
|  | CLCN5 | Chloride voltage-gated channel 5 |
|  | AR | Androgen receptor |
|  | ZNF75D | Zinc finger protein 75D |
|  | NHSL2 | NHS like 2 |
|  | NHS | NHS actin remodeling regulator |
|  | DUSP9 | Dual specificity phosphatase 9 |
|  | MID1IP1 | MID1 interacting protein 1 |
|  | TSC22D3 | TSC22 domain family member 3 |
|  | LONRF3 | LON peptidase N-terminal domain and ring finger 3 |
|  | HTR3B | 5-hydroxytryptamine receptor 3B |
|  | HTR3C | 5-hydroxytryptamine receptor 3C |
|  | HTR3D | 5-hydroxytryptamine receptor 3D |
|  | HTR3E | 5-hydroxytryptamine receptor 3E |
|  | UCP2 | Uncoupling protein 2 |
|  | NRCAM | Neuronal cell adhesion molecule |
|  | IMMP2L | Inner mitochondrial membrane peptidase subunit 2 |
|  | HOXB8 | Homeobox B8 |
|  | PRL | Prolactin |
|  | CYP3A4 | Cytochrome P450 family 3 subfamily A member 4 |
|  | CAT | Catalase |
|  | SLC22A2 | Solute carrier family 22 member 2 |
|  | ABCB1 | ATP binding cassette subfamily B member 1 |
|  | TH | Tyrosine hydroxylase |
|  | KCNH2 | Potassium voltage-gated channel subfamily H member 2 |
|  | ARC | Activity regulated cytoskeleton associated protein |
|  | HMGCS1 | 3-hydroxy-3-methylglutaryl-CoA synthase 1 |
|  | EGR1 | Early growth response 1 |
|  | POMC | Proopiomelanocortin |
|  | NPY | Neuropeptide Y |
|  | NR3C1 | Nuclear receptor subfamily 3 group C member 1 |
|  | INHA | Inhibin subunit alpha |
|  | SOD1 | Superoxide dismutase 1 |
|  | BCL2 | BCL2 apoptosis regulator |
|  | HOMER1 | Homer scaffold protein 1 |
|  | CASP3 | Caspase 3 |
|  | CTNNB1 | Catenin beta 1 |
|  | MAPK1 | Mitogen-activated protein kinase 1 |
|  | KCNJ3 | Potassium inwardly rectifying channel subfamily J member 3 |
|  | KCNJ6 | Potassium inwardly rectifying channel subfamily J member 6 |
|  | SLC18A2 | Solute carrier family 18 member A2 |
|  | NUPR1 | Nuclear protein 1, transcriptional regulator |
|  | CDK5 | Cyclin dependent kinase 5 |
|  | FOSB | FosB proto-oncogene, AP-1 transcription factor subunit |
|  | VEGFA | Vascular endothelial growth factor A |
|  | JUN | Jun proto-oncogene, AP-1 transcription factor subunit |
|  | PENK | Proenkephalin |
|  | FNDC4 | Fibronectin type III domain containing 4 |
|  | SYP | Synaptophysin |
|  | CRH | Corticotropin releasing hormone |
|  | KCNJ5 | Potassium inwardly rectifying channel subfamily J member 5 |
|  | MAP2 | Microtubule associated protein 2 |
|  | GDPD3 | Glycerophosphodiester phosphodiesterase domain containing 3 |
|  | BAX | BCL2 associated X, apoptosis regulator |
|  | AKT1 | AKT serine/threonine kinase 1 |
|  | IL6 | Interleukin 6 |
|  | GDNF | Glial cell derived neurotrophic factor |
|  | INHBE | Inhibin subunit beta E |
|  | CREB1 | cAMP responsive element binding protein 1 |
|  | CASP7 | Caspase 7 |
|  | SLC2A3 | Solute carrier family 2 member 3 |
|  | TAGLN | Transgelin |
|  | CAMK4 | Calcium/calmodulin dependent protein kinase IV |
|  | CYP2C8 | Cytochrome P450 family 2 subfamily C member 8 |
|  | EGR2 | Early growth response 2 |
|  | NR0B2 | Nuclear receptor subfamily 0 group B member 2 |
|  | C10ORF10 | DEPP1 autophagy regulator |
|  | SERPINA3 | Serpin family A member 3 |
|  | GSTP1 | Glutathione S-transferase pi 1 |
|  | MAPK3 | Mitogen-activated protein kinase 3 |
|  | HTR7 | 5-hydroxytryptamine receptor 7 |
|  | GABRA1 | Gamma-aminobutyric acid type A receptor subunit alpha1 |
|  | DUSP1 | Dual specificity phosphatase 1 |
|  | DBH | Dopamine beta-hydroxylase |
|  | GRIA3 | Glutamate ionotropic receptor AMPA type subunit 3 |
|  | FAS | Fas cell surface death receptor |
|  | FABP1 | Fatty acid binding protein 1 |
|  | CYP3A5 | Cytochrome P450 family 3 subfamily A member 5 |
|  | SLC1A1 | solute carrier family 1 member 1 |
|  | HTR2A | 5-hydroxytryptamine receptor 2A |
|  | DRD2 | Dopamine receptor D2 |
|  | DLGAP3 | DLG associated protein 3 |

| Supplementary.Table.3 Common genes between Schizophrenia and Obsessive-compulsive disorder associated genes. | | |
| --- | --- | --- |
| Index | **Genes symbol** | **Gene full name** |
|  | SLC6A4 | Solute carrier family 6 member 4 |
|  | GRIK2 | Glutamate ionotropic receptor kainate type subunit 2 |
|  | MAOA | Monoamine oxidase A |
|  | DRD4 | Dopamine receptor D4 |
|  | COMT | Catechol-O-methyltransferase |
|  | GRIN2B | Glutamate ionotropic receptor NMDA type subunit 2B |
|  | BDNF | Brain derived neurotrophic factor |
|  | HTR1B | 5-hydroxytryptamine receptor 1B |
|  | TPH2 | Tryptophan hydroxylase 2 |
|  | OLIG2 | Oligodendrocyte transcription factor 2 |
|  | GABBR1 | Gamma-aminobutyric acid type B receptor subunit 1 |
|  | NTRK2 | Neurotrophic receptor tyrosine kinase 2 |
|  | MOG | Myelin oligodendrocyte glycoprotein |
|  | DRD3 | Dopamine receptor D3 |
|  | ESR1 | Estrogen receptor 1 |
|  | NTRK3 | Neurotrophic receptor tyrosine kinase 3 |
|  | HTR3A | 5-hydroxytryptamine receptor 3A |
|  | LMX1A | LIM homeobox transcription factor 1 alpha |
|  | mTOR | Mechanistic target of rapamycin kinase |
|  | SLC18A1 | Solute carrier family 18 member A1 |
|  | GAD1 | Glutamate decarboxylase 1 |
|  | GAD2 | Glutamate decarboxylase 2 |
|  | SLC6A3 | Solute carrier family 6 member 3 |
|  | GRIK3 | Glutamate ionotropic receptor kainate type subunit 3 |
|  | SLC1A2 | Solute carrier family 1 member 2 |
|  | ESR2 | Estrogen receptor 2 |
|  | OPRM1 | Opioid receptor mu 1 |
|  | NRXN1 | Neurexin 1 |
|  | KCNH5 | Potassium voltage-gated channel subfamily H member 5 |
|  | NRXN3 | Neurexin 3 |
|  | OXTR | Oxytocin receptor |
|  | TPH1 | Tryptophan hydroxylase 1 |
|  | NTRK1 | Neurotrophic receptor tyrosine kinase 1 |
|  | NFKBIL1 | NFKB inhibitor like 1 |
|  | CYP2D6 | Cytochrome P450 family 2 subfamily D member 6 |
|  | GABRG2 | Gamma-aminobutyric acid type A receptor subunit gamma2 |
|  | NOS1AP | Nitric oxide synthase 1 adaptor protein |
|  | CNTNAP2 | Contactin associated protein 2 |
|  | NPSR1 | Neuropeptide S receptor 1 |
|  | GRIA2 | Glutamate ionotropic receptor AMPA type subunit 2 |
|  | Grm5 | Glutamate metabotropic receptor 5 |
|  | CYP2E1 | Cytochrome P450 family 2 subfamily E member 1 |
|  | DLG4 | Discs large MAGUK scaffold protein 4 |
|  | HTR1A | 5-hydroxytryptamine receptor 1A |
|  | RGS4 | Regulator of G protein signaling 4 |
|  | MAOB | Monoamine oxidase B |
|  | CYP2C19 | Cytochrome P450 family 2 subfamily C member 19 |
|  | DRD1 | Dopamine receptor D1 |
|  | ANKK1 | Ankyrin repeat and kinase domain containing 1 |
|  | HLA-DRB1 | Major histocompatibility complex, class II, DR beta 1 |
|  | AR | Androgen receptor |
|  | HTR3B | 5-hydroxytryptamine receptor 3B |
|  | HTR3C | 5-hydroxytryptamine receptor 3C |
|  | HTR3D | 5-hydroxytryptamine receptor 3D |
|  | HTR3E | 5-hydroxytryptamine receptor 3E |
|  | UCP2 | Uncoupling protein 2 |
|  | NRCAM | Neuronal cell adhesion molecule |
|  | PRL | Prolactin |
|  | CYP3A4 | Cytochrome P450 family 3 subfamily A member 4 |
|  | ABCB1 | ATP binding cassette subfamily B member 1 |
|  | TH | Tyrosine hydroxylase |
|  | KCNH2 | Potassium voltage-gated channel subfamily H member 2 |
|  | ARC | Activity regulated cytoskeleton associated protein |
|  | EGR1 | Early growth response 1 |
|  | POMC | Proopiomelanocortin |
|  | NPY | Neuropeptide Y |
|  | NR3C1 | Nuclear receptor subfamily 3 group C member 1 |
|  | SOD1 | Superoxide dismutase 1 |
|  | HOMER1 | Homer scaffold protein 1 |
|  | CTNNB1 | Catenin beta 1 |
|  | SLC18A2 | Solute carrier family 18 member A2 |
|  | SYP | Synaptophysin |
|  | CRH | Corticotropin releasing hormone |
|  | MAP2 | Microtubule associated protein 2 |
|  | AKT1 | AKT serine/threonine kinase 1 |
|  | IL6 | Interleukin 6 |
|  | GDNF | Glial cell derived neurotrophic factor |
|  | CREB1 | cAMP responsive element binding protein 1 |
|  | EGR2 | Early growth response 2 |
|  | GSTP1 | Glutathione S-transferase pi 1 |
|  | HTR7 | 5-hydroxytryptamine receptor 7 |
|  | GABRA1 | Gamma-aminobutyric acid type A receptor subunit alpha1 |
|  | DBH | Dopamine beta-hydroxylase |
|  | GRIA3 | Glutamate ionotropic receptor AMPA type subunit 3 |
|  | FAS | Fas cell surface death receptor |
|  | CYP3A5 | Cytochrome P450 family 3 subfamily A member 5 |
|  | SLC1A1 | Solute carrier family 1 member 1 |
|  | HTR2A | 5-hydroxytryptamine receptor 2A |
|  | DRD2 | Dopamine receptor D2 |
